# Structuring heterogeneous biological information using fuzzy clustering of k-partite graphs

**DOI:** 10.1186/1471-2105-11-522

**Published:** 2010-10-20

**Authors:** Mara L Hartsperger, Florian Blöchl, Volker Stümpflen, Fabian J Theis

**Affiliations:** 1 Institute of Bioinformatics and Systems Biology (MIPS), Helmholtz Zentrum München - German Research Center for Environmental Health, Ingolstädter Landstr. 1, 85764 Neuherberg, Germany; 2 Department of Mathematical Science, Technische Universität München, Boltzmannstr. 3, 85748 Garching, Germany

## Abstract

**Background:**

Extensive and automated data integration in bioinformatics facilitates the construction of large, complex biological networks. However, the challenge lies in the interpretation of these networks. While most research focuses on the unipartite or bipartite case, we address the more general but common situation of *k*-partite graphs. These graphs contain *k *different node types and links are only allowed between nodes of different types. In order to reveal their structural organization and describe the contained information in a more coarse-grained fashion, we ask how to detect clusters within each node type.

**Results:**

Since entities in biological networks regularly have more than one function and hence participate in more than one cluster, we developed a *k*-partite graph partitioning algorithm that allows for overlapping (fuzzy) clusters. It determines for each node a degree of membership to each cluster. Moreover, the algorithm estimates a weighted *k*-partite graph that connects the extracted clusters. Our method is fast and efficient, mimicking the multiplicative update rules commonly employed in algorithms for non-negative matrix factorization. It facilitates the decomposition of networks on a chosen scale and therefore allows for analysis and interpretation of structures on various resolution levels. Applying our algorithm to a tripartite disease-gene-protein complex network, we were able to structure this graph on a large scale into clusters that are functionally correlated and biologically meaningful. Locally, smaller clusters enabled reclassification or annotation of the clusters' elements. We exemplified this for the transcription factor MECP2.

**Conclusions:**

In order to cope with the overwhelming amount of information available from biomedical literature, we need to tackle the challenge of finding structures in large networks with nodes of multiple types. To this end, we presented a novel fuzzy *k*-partite graph partitioning algorithm that allows the decomposition of these objects in a comprehensive fashion. We validated our approach both on artificial and real-world data. It is readily applicable to any further problem.

## Background

With the increasing availability of high throughput "-omics" technologies such as next generation sequencing, proteomics or metabolic profiling an enormous amount of textual data is accessible in the biomedical literature. Hence, methods able to structure these heterogeneous data and to extract new knowledge gain more and more importance. Learning approaches often focus on the analysis of homogeneous data sets that can be represented as graphs having vertices of a single type only. However, biological networks are complex and highly diverse and therefore often involve objects of multiple types, forming *k*-partite graphs consisting of different kinds of vertices. We use this representation as it provides a more comprehensive picture of the underlying structure compared to the widely used graph transformations. These so-called projections - e.g. of a bipartite network into an unipartite version - discard important information [1]. For instance, [2] shows that in the case of metabolism the use of projections leads to wrong interpretations of some of the most relevant graph attributes, whereas the bipartite view offers a cleaner interpretation of its topological features.

The human disease network presented in [3] is an example for a bipartite graph having two disjoint sets of vertices. Here, structural questions need to be addressed outside of the unipartite graph setting. One set of nodes represents all known genetic disorders, the vertices of the other partition correspond to all known disease genes in the human genome. A disorder and a gene are connected if mutations in that gene are implicated in that disorder. Other examples of bipartite networks are protein complex or gene-localization, gene-function or microRNA-target networks. The integration of such network data then leads to complex *k*-partite graphs.

A key question is how to interpret the internal organization of these networks. A possible answer may be a modular decomposition, which implies the coexistence of structural subunits associated with more highly interconnected parts. We regard the identification of these a priori unknown building blocks - such as for instance functional modules in protein-protein-interaction (PPI) networks - as clustering methods. The clusters and their interconnections are essential for understanding the underlying functional properties. They structure biological data by compressing their information into a condensed form.

Most available clustering methods do not treat the single node types (partitions) separately and therefore do not represent the global cluster structure of *k*-partite networks correctly. While this has been addressed in terms of algorithms for some time now [4-6], not many applications were successfully implemented in bioinformatics yet, although the field commonly deals with such networks [1]. A particular issue that may hamper application to biological data is that most existing algorithms identify separated, disjoint clusters by assigning each point to exactly one cluster [7,8]. This is unrealistic for biological systems as e.g. genes or proteins commonly participate in multiple processes or pathways [9]. So far, only a few approaches exist that allow the detection of overlapping clusters. These either assign each data point to several hard clusters [10] or determine a degree of membership to each cluster [11,12]. Such methods are known as fuzzy clustering, but have not been applied to the common biological case of *k*-partite graphs.

To overcome these difficulties we developed a novel fuzzy clustering algorithm based on a non-negative matrix factorization (NMF) model [13]. Our algorithm extends a hard clustering algorithm recently put forward in [14]. This algorithm clusters each node type of the graph separately and then connects clusters via a smaller, weighted *k*-partite graph in an alternating minimization procedure. Thereby, the cluster assignment in the first step is made in a binary fashion. This disjoint clustering is a feature that is often achieved by soft clustering algorithms when not forcing explicit cluster overlap [11]. However, it can be easily seen that the cost function proposed is not fully minimized. Our computationally efficient and scalable algorithm avoids this problem. It is similar in structure to multiplicative algorithms for NMF, with the difference that we address a three-matrix factorization problem (see e.g. [15]), and have to deal with a multi-summand cost function. As our cost function is monotonous with respect to the number of clusters, our algorithm allows detection of clusters on different scales. Hence, we are able to decompose the network on different resolution levels.

The manuscript is organized as follows. In the first part, we develop the fuzzy clustering algorithm and validate it on a toy example and graphs with known modular structure. Then, we apply it to a tripartite disease-gene-protein complex graph representing an expanded view of the human disease network from [3] extended by protein complexes [16]. By integrating functional annotation we demonstrate that we are able to structure this complex graph into biologically meaningful clusters on a large scale. Finally, focusing on the small-scale architecture, we identify overlapping clusters that give a more comprehensive picture about gene-disease connections rather than looking at disjoint clusters alone. We exemplify how this clustering allows for reclassification, annotation or even detection of misclassified elements on a local level.

## Results and Discussion

A *k*-partite graph is a graph *G *= (*V*, *E*) of edges *E *between a set of vertices *V *together with a partition of the vertices into *k *disjoint subsets *V_i _*such that no two vertices in the same subset are adjacent. For *k *= 1 this reduces to the standard graph, where we do not take into account different node types. Graphs with two partitions are called bipartite. Let *n_i _*:= |*V_i_*| be the number of vertices in the partition *i*, *i *= 1 ... *k*. We represent the graph as a set of *n_i _*× *n_j_*-dimensional adjacency matrices **A**^(*ij*) ^for all *i*, *j *with 1 ≤ *i *<*j *≤ *k*. Typically, each matrix element is either 0 or 1, but we only restrict the matrices to have non-negative coefficients thereby allowing weighted graphs as well.

### Approach

We want to approximate *G *by a smaller *k*-partite cluster network *H *which we call *backbone network*. It is defined on the fuzzy clusters of each *G*-partition *V_i_*. We fix the number of clusters in partition *i *to *m_i_*. We denote a non-negative *n_i _*× *m_i_*-dimensional matrix **C**^(*i*) ^to be a *fuzzy clustering *of *V_i_*, if it is right-stochastic, i.e. $\sum_{l=1}^{m_{i}}C_{kl}^{\left(i\right)}=1$ for all *k*. Its (*k*, *l*)-th element $C_{kl}^{\left(i\right)}$ gives the degree of membership of the original node *k *to the backbone node *l*.

**Figure 1:**
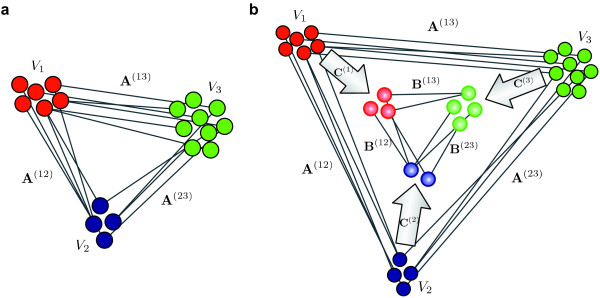
**Illustration of the fuzzy clustering approach**. We want to approximate the tripartite example graph *G *in **(a) **by a smaller tripartite cluster network *H*, the so-called *backbone graph ***(b)**. The decomposition into fuzzy clusters connected by this backbone must explain the original connectivity as good as possible. The edges of *G *are collected in adjacency matrices **A**^(*ij*) ^connecting the elements of the partitions *i *and *j*. The approximation of *G *by the backbone graph is encoded in the adjacency matrices **B**^(*ij*) ^connecting the fuzzy node clusters **C**^(*i*)^. These matrices **C**^(*i*) ^collect the degrees of membership of each node of partition *V_i _*to each cluster of this type. Its (*k*, *l*)-th element $C_{kl}^{\left(i\right)}$ specifies how much node *k *belongs to the backbone node *l*.

Then we search for a *k*-partite graph *H *with *m_i _*× *m_j _*adjacency matrices **B**^(*ij*) ^and a fuzzy clustering *C *:= (**C**^(*i*)^)_*i *= 1,...,*k *_such that the connectivity explained by *H *is as close as possible to *G *after clustering according to *C*. Figure 1 shows an example graph and its approximation by a backbone network. From the approximation, we can easily reconstruct an edge $A_{uv}^{\left(ij\right)}$ between two nodes *u *and *v *from partitions *i *and *j *in the original graph. To this end, we have to sum up all edge weights **B**^(*ij*) ^in the backbone graph that connect the communities *u *and *v *are assigned to. Of course, in a fuzzy environment these contributions have to be weighted by the nodes' degrees of membership **C**^(*i*) ^and **C**^(*j*)^, respectively. Taken together, the entry of the adjacency matrix can be reconstructed as the double sum

$$A_{uv}^{\left(ij\right)}\approx \sum_{x=1}^{m_{i}}\sum_{y=1}^{m_{j}}C_{ux}^{\left(i\right)}B_{xy}^{\left(ij\right)}C_{vy}^{\left(j\right)}.$$

Writing this in matrix notation, we see that the requirement of explaining maximum possible connectivity means that the adjacency matrices **A**^(*ij*) ^are best possible approximated by factorizations of the form

$$A^{\left(ij\right)}\approx C^{\left(i\right)}B^{\left(ij\right)}{\left(C^{\left(j\right)}\right)}^{⊤}.$$

We can measure the difference between the two graphs *H *and *G *in a variety of ways. In [14], this choice has been circumvented by focusing on arbitrary Bregman divergences, see e.g. [17], which still allow efficient reformulation of gradient-type algorithms without knowing the specific formula. This is also possible in our case of multiplicative update rules, as has been shown for NMF by [15]. Here, we choose the minimum square distance as the cost function. This implies minimization of

$$f(H,C):=\sum_{i<j}{\left\|A^{\left(ij\right)}-C^{\left(i\right)}B^{\left(ij\right)}{\left(C^{\left(j\right)}\right)}^{⊤}\right\|}_{F}^{2},$$

where ${\left\|.\right\|}_{F}^{2}$ denotes the squared Frobenius norm i.e. the square sum of the matrix elements. This cost function is obviously monotonous with respect to the number of clusters in each partition.

#### Algorithm formulation and relation to other work

We aim at minimizing the cost function *f*(*H*, *C*) using a local algorithm extending gradient descent. In order to avoid the choice of update rates and to ensure positivity of both the backbone network and the degrees of membership of all nodes, we employ multiplicative update rules. This strategy is widely used in algorithms for non-negative matrix factorization (NMF) [18]. We find the following update rules (see Methods for the detailed derivation):

$$\begin{array}{l} B_{rs}^{\left(ij\right)}\leftarrow B_{rs}^{\left(ij\right)}\frac{{\left({\left(C^{\left(i\right)}\right)}^{⊤}A^{\left(ij\right)}C^{\left(j\right)}\right)}_{rs}}{{\left({\left(C^{\left(i\right)}\right)}^{⊤}C^{\left(i\right)}B^{\left(ij\right)}{\left(C^{\left(j\right)}\right)}^{⊤}C^{\left(j\right)}\right)}_{rs}}\\ C_{rs}^{\left(i\right)}\leftarrow C_{rs}^{\left(i\right)}\frac{{\left(\sum_{j\neq i}A^{\left(ij\right)}C^{\left(j\right)}{\left(B^{\left(ij\right)}\right)}^{⊤}\right)}_{rs}}{{\left(\sum_{j\neq i}C^{\left(i\right)}B^{\left(ij\right)}{\left(C^{\left(j\right)}\right)}^{⊤}C^{\left(j\right)}{\left(B^{\left(ij\right)}\right)}^{⊤}\right)}_{rs}} \end{array}$$

We note that these update rules do not increase the cost function (1). This can be shown via auxiliary functions similar to NMF [18] and multi-factor NMF [15], which has been applied in a related model for co-clustering of microarray data [19]. This theoretical result implies convergences of the update rules. However, in contrast to early statements in NMF [18], it does not necessarily imply convergence to stationary points of the Euclidean norm (zero of the differential from (1)), since the update steps may be too small to reach those points. Another possible drawback of such multiplicative updates is the fact that once a matrix entry has been set to zero (which may happen due to zeros in **A**^(*ij*) ^or to numerics), the coefficient will never then be able to become positive again during learning. This is one of the reasons, why sometimes alternating least-squares algorithms are chosen [20].

**Figure 2:**
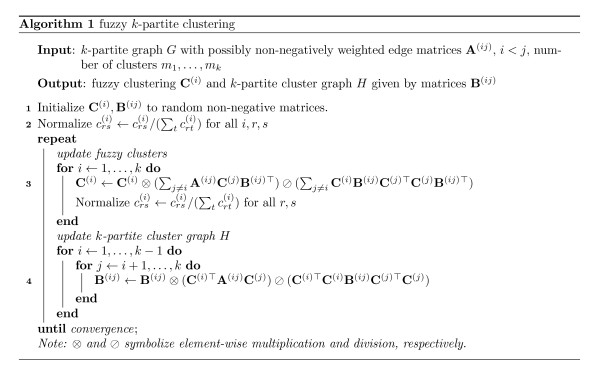
**Fuzzy clustering algorithm**. Summarization of the final fuzzy *k*-partite clustering algorithm.

We have not yet taken into account the constraint that the fuzzy clusterings **C**^(*i*) ^are required to be right-stochastic. We force this constraint by regularly projecting each row of **C**^(*i*) ^onto the sphere of the 1-norm. The final fuzzy *k*-partite clustering algorithm is summarized in Figure 2. Implementations are available as Additional Files 1 and 2.

Our algorithm has two nested loops over the number of partitions *k*, hence its runtime depends quadratically on this number. The update rules for **C**^(*i*) ^and **B**^(*ij*) ^however are fully vectorized and contain only matrix operations with non-square matrices. Their time complexity is dominated by the occurring matrix products: multiplying two matrices of sizes *s*_1 _× *s*_2 _and *s*_2 _× *s*_3 _is of complexity O(*s*_1_*s*_2_*s*_3_). Assuming that the cluster numbers *m_i _*are smaller than the largest two partition sizes, the total time complexity of the fuzzy clustering algorithm can then be estimated as

$$\#\text{iterations}\times O(k^{\text{2}}n_{\text{max1}}n_{\text{max2}}m_{\text{max}}).$$

Here, *n*_max1 _and *n*_max2 _denote the sizes of the largest and the second-largest partition, *m*_max _is the maximum number of clusters within any partition. Hence, the runtime grows only quadratically in the total number of nodes in the case of graphs with similarly large partitions. In general, the runtime is linear in each partition's size *n_i _*and cluster number *m_i_*. We additionally confirmed this theoretical analysis by simulations shown in Additional File 3.

#### Algorithm evaluation - Toy example

**Figure 3:**
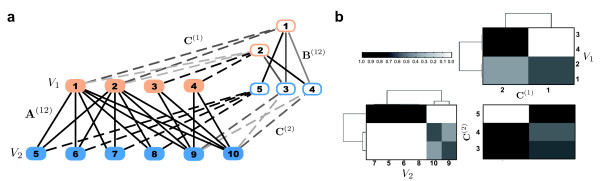
**Illustration of the cluster decomposition of a bipartite toy example**. **(a) **We demonstrate the graph decomposition with our algorithm on a small bipartite graph with overlapping cluster structure. The original graph consists of partitions *V*_1 _= {1 ... 4} (red filled nodes) and *V*_2 _= {5 ... 10} (blue filled nodes) connected by edges **A**^(12) ^colored in black. We decomposed it into two clusters for partition *V*_1 _and three clusters for partition *V*_2_. The resulting fuzzy clustering is illustrated as a weighted graph connecting original nodes to cluster nodes (framed red and blue). The cluster assignments **C**^(1) ^and **C**^(2) ^are indicated by dashed lines, where the coloring corresponds to the degree of cluster membership. The interconnections of the clusters form the *backbone graph*, encoded in the adjacency matrix **B**^(12) ^which we denote by continous lines where color indicates the edge weight. Another way of illustrating the graph decomposition is shown in **(b)**. It is clearer especially for larger graphs. First, we plot hierarchical clusterings of the nodes' degrees of membership in partitions *V*_1 _and *V*_2 _(encoded by **C**^(1) ^and **C**^(2)^). This facilitates the identification of overlapping clusters (e.g. nodes 1 and 10 are assigned to more than one cluster) or hard cluster assignments (e.g. node 5). The backbone graph **B**^(12) ^is shown bottom right. This backbone graph is densely connected in our example.

For illustration, we applied our algorithm to a bipartite graph having several vertices connected with all vertices of the other partition (e.g. nodes 1 and 10). Figure 3 shows that these vertices are assigned to two clusters with distinct degree of membership, whereas vertices partially connected are element of a single cluster only (e.g. 3). This demonstrates the idea and importance of using a fuzzy that allows for overlapping clusters.

#### Algorithm evaluation - Performance analysis

Before applying our algorithm to real-world data, we tested its behavior on simulated data with controlled cluster structure. In particular, we compared it to the hard clustering algorithm from [14]. We used exactly the same stopping criteria for both algorithms. We generated random, modularly structured *k*-partite networks as described in the Methods section. In order to compare algorithm performance, we determined runtime, final cost function value and the quality of cluster estimation in four different settings. We restricted ourselves to bipartite and layered tripartite graphs with two different noise settings because Long *et al. *provided code for analyzing these special cases only.

**Figure 4:**
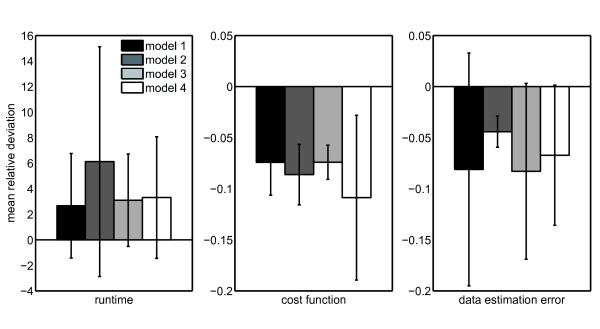
**Performance on toy models**. We validated our algorithm on graphs with predefined cluster structure. To this end, we compared it with the hard clustering method by Long *et al. *on four different random toy models, see Table 1. The plot shows the mean relative deviation between the two algorithms relative to the results of the hard clustering. Error bars denote standard deviations over 1000 runs. We see that the fuzzy cluster assignments of our method require much more runtime, but both cost function and data estimation error (see Methods) are significantly smaller. The large standard deviations show the dependency of the decomposition on the random initial conditions. Therefore, by default we perform multiple restarts with different initializations.

We found that while the method of Long *et al. *performed around two times faster, our algorithm produced around 10% lower cost function and was able to estimate the cluster structure better (see Figure 4). This difference in algorithm runtime originates from the much more fine-tuning of the continuous degrees of membership compared to hard cluster assignments. These require less update steps until convergence.

#### Algorithm evaluation - Stability of clusters

In contrast to deterministic methods like for instance singular value decomposition (SVD), NMF-based methods have problems concerning robust computation. Even for standard unipartite NMF there is no unique global minimum of the cost function [21]. Our algorithm aims to minimize the cost function using a local optimization strategy extending gradient descent. This implies that the algorithm only converges to a local minimum. The algorithm is indeterministic, it does not converge to the same solution on each run due to the stochastic nature of initial conditions. Thus, following the general proceeding in literature on NMF [21,22], we compare the local minima from several different starting points (multiple restarts), using the results of the best local minimum found.

In order to illustrate the stability of the fuzzy clustering algorithm we applied it to a toy network with well defined cluster structure using multiple restarts. We compared the clustering results of 100 runs and quantified the cluster stability using a fuzzy variant of the rand index recently proposed in [23]. As we show in Additional File 4, our algorithm is able to reproduce the true clustering results in more than 70% of the runs. Hence, we can not guarantee that the local optimization finds a global minimum of the cost function, and with this the cluster structure of a graph. This illustrates the critical need for multiple restarts.

### Structuring biological data

**Figure 5:**
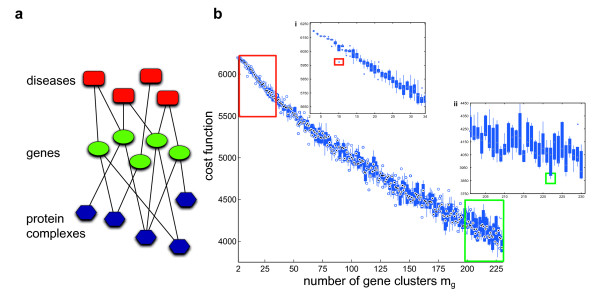
**Decomposition of a gene-disease-protein complex network**. We integrated the gene-disease network from [3] with human protein complexes from the CORUM database [16]. This resulted in a layered tripartite graph, which is schematically drawn in **(a)**. We performed a 10-fold approximation of this graph to estimate appropriate numbers of clusters. The boxplot curve **(b) **shows how the cost function *f*(*H*, *C*) from equation (1) depends on the number of gene clusters *m_g_*. The true minima of the cost function are decreasing with *m_g_*, and this is also visible in the approximated minima using our proposed algorithm. Therefore, we are able to identify structures on various resolution levels. The details represent the cost function course for large-scale clustering **(i) **and a decomposition on small scale **(ii)**, respectively. For our detailed analyses, we used the decompositions showing steep drops in the cost function marked by the red and green boxes.

In order to exemplify the analysis of biological networks, we applied our algorithm to a layered tripartite disease-gene-protein complex network, see Figure 5a for an illustration. In this graph, a disorder and a gene are connected if mutations in that gene are implicated in that disorder. A complex and a gene are linked if the gene is coding for a protein part of the complex. We constructed this graph by integrating the human gene-disease network from [3] and protein complexes from the CORUM database, as explained in the Methods section.

An important feature of many biological networks is their hierarchical organization, where higher-level structure is composed of multiple instances of a lower-level structures of different types [24]. This implies that small groups of nodes organize in a hierarchical manner to increasingly large groups on many different scales [25,26]. To account for this topological characteristic we have to be able to extract relevant information on an appropriate, pre-defined resolution level.

We addressed this issue by analyzing the very global structure and a detailed local level of the disease-gene-protein complex network. In the following, we first present the results of a decomposition into large clusters which demonstrates that our method is generally applicable to biological data. Then, we discuss smaller clusters that allowed for a precise interpretation of single elements.

As discussed before, due to its random initialization our algorithm is inherently indeterministic. Different clustering results have of course a significant impact on the interpretation of the biological meaning of the results. We show in Additional File 4 that our algorithm is quite stable on graphs with well defined cluster structure. To avoid analyzing a local minimum, we discuss performance over 10 runs and verify that the disease-gene-protein complex network has indeed a defined cluster structure in Additional File 5.

Dealing with a theoretically monotonous cost function, it is hard to determine the optimal numbers of clusters for each node type in which the graph has to be partitioned. Appropriate values are not apparent from prior knowledge about our data set. We therefore chose desired approximate resolutions *m_g _*for the gene partition. The number of clusters *m_c _*and *m_d _*in the protein complex and disease partitions were then scaled according to their partitions' sizes (see Methods). We use this heuristics, since a brute-force sampling of the three-dimensional parameter space is computationally out of reach. Then, we looked for plateaus and steep drops in the cost function within a certain range around this value *m_g _*and chose a local optimum of the algorithmically found decompositions. In Additional File 5 we performed simulations showing that the profile of the cost function may indeed indicate for a proper number of clusters in graphs with known cluster structure.

#### Large-scale clustering

**Figure 6:**
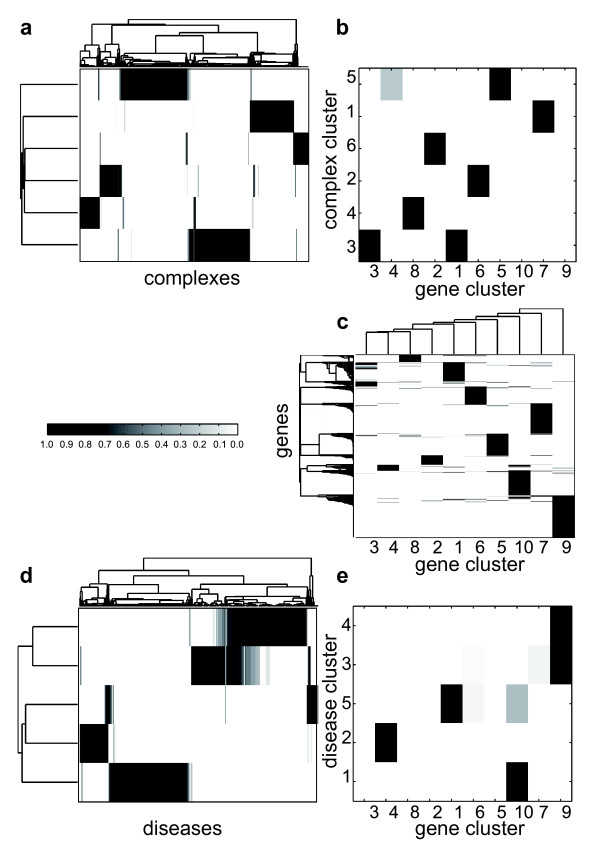
**Illustration of large-scale cluster structures in the gene-disease-protein complex network**. The large-scale decomposition of the gene-disease-protein complex network is illustrated as described in Figure 2b. The hierarchical clustering of the nodes' degrees of membership of the **(a) **complex, **(c) **gene and the **(d) **disease partition show that the majority of elements was assigned to single clusters. However, a considerable amount of cluster overlaps exists, e.g. for the disease clusters 3 and 4. The backbones for gene-complex **(b) **and for gene-disease **(e) **are sparsely connected, but show that locally overlapping clusters tend to interconnect with the same clusters of the other partition; e.g. disease cluster 3 and 4 are both connected to gene cluster 9 with large weights.

First, we focused on the identification of large clusters. Figure 5b shows the distribution of the cost function values after algorithm convergence for each parameter setting. In the following discussion, we used (*m_g_*, *m_c_*, *m_d_*) = (10, 5, 6) as it showed the first steep drop in the cost function. Moreover, here we observed a significant local minimum of the cost function values of the algorithmically determined decompositions. From the illustration of the decomposition in Figure 6 we see that the resulting clusters vary strongly in size. For all partitions, the majority of elements was assigned to a single cluster with degree of membership *μ *> = 0.9. This demonstrates that the analyzed graph has a well defined cluster structure at the desired resolution level. The corresponding histograms are given in Additional File 5. Therein we also discuss an example illustrating that such large degrees of membership are rarely found in graphs lacking any cluster structure. However, there exists also a considerable amount of elements assigned to several clusters simultaneously, e.g. in complex clusters 3 and 5, gene clusters 1 and 3 or disease clusters 3 and 4. This confirms the usefulness of our fuzzy approach.

##### Cluster evaluation

To determine whether the resulting clusters are biologically reasonable, we applied GO enrichment analysis (see Methods) to the clusters of the gene partition. We found, for instance, that for the genes in the two overlapping clusters 1 and 3 significantly enriched GO-terms are *cell cycle *and *cellular response to stimulus/stress*. Genes in cluster 4 can be related to e.g. *death*, *cell proliferation *and *developmental processes*, whereas cluster 6 represents *translation*. *Gene expression*-associated GO-terms such as *RNA processing *and *splicing *were detected in cluster 7. This shows that our method was able to identify biologically meaningful functionally enriched clusters. The complete tables for GO enrichments in all clusters are shown in Additional File 6.

The interconnectivity of the in total 21 clusters is sparse (see Figure 6). The skeleton for the global cluster structure for both underlying bipartite graphs (gene-complex and gene-disease) demonstrates that locally overlapping clusters also tend to interconnect with the same clusters of the other partition; for instance, disease clusters 3 and 4 are both connected with gene cluster 9. To evaluate the extracted backbone graph, in the following we tested the hypothesis that interconnected clusters of different partitions are also functionally correlated.

##### Gene-complex interconnections

**Figure 7:**
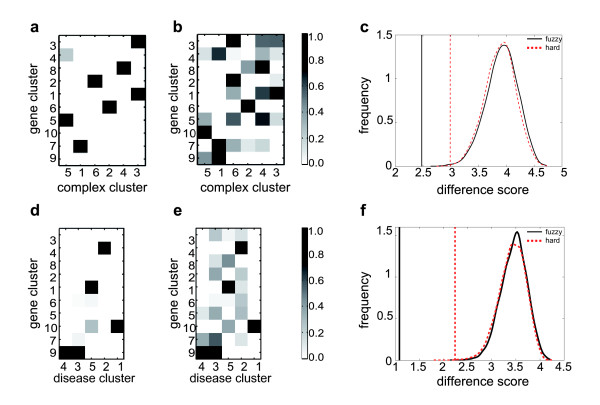
**Evaluation of the backbone of the gene-disease-protein complex network**. To evaluate the large-scale clustering we additionally included functional annotations. **(a) **and **(b) **compare the gene-complex backbone graph with the functional correlations of the extracted clusters according to FunCat annotation. Similarly, **(d) **and **(e) **show the gene-disease backbone and the clusters' disorder class correlations (see Methods). We see that interconnected clusters also seem to correlate in their annotations. To test this hypothesis rigorously, we calculated difference scores as defined in Methods in order to quantify the correlation of the backbones and their annotations, respectively. Vertical lines in **(c) **and **(f) **correspond to these difference scores for the fuzzy (black) and the hard (red) clustering. Comparing these values to the difference scores for 10^5 ^randomized cluster assignments we obtain significant *p*-values, both < 10^-5^. The correlations between annotations of connected clusters of the backbone is higher when applying the fuzzy approach.

Assuming that the resulting interconnected gene and complex clusters are functionally related, one expects to see a similar profile for FunCat annotation and backbone interconnectivity of each cluster. This hypothesis was verified in Figure 7, where for instance complex cluster 3 and the interconnected gene clusters 1 and 3 show a high binary FunCat correlation. The difference score (as defined in Methods) between backbone interconnectivity and annotation correlation is 2.48, resulting in a *p*-value < 10^-5^. To compare the results of the fuzzy clustering approach with the results for the disjoint clustering method from [14] we applied the algorithm with the same parameter settings and identical annotation and randomization procedure to the obtained clusters. For hard clustering we achieved a larger difference score of 2.99 which corresponds to a significant *p*-value of 0.0015.

##### Gene-disease interconnections

To ascertain that our method is able to detect biological feasible clusters in all partitions, we determined for each gene and disease cluster disorder class profiles. Again, we observed a high similarity between backbone interconnectivity and disorder correlation having a difference score of 1.09 (*p*-value < 10^-5^). For instance, gene cluster 1 and 10 and the interconnected disease clusters 1 and 5 show a high disorder correlation (see Figure 7).

#### Small-scale clustering

We showed that our method is able to both detect and interconnect biologically meaningful clusters. However, due to their size of about 279 genes on average the single clusters are hard-to-interpret. The detection of smaller clusters representing biological units enables a precise biological interpretation. In the following, we describe results for (*m_g_*, *m_c_*, *m_d_*) = (222, 135, 112), where we found the lowest value of the cost function (see Figure 5b). This setting accounts for an average cluster size of 10 genes.

In order to make use of the cluster overlaps, we looked for genes assigned to more than one cluster with a degree of membership of *μ *> 0.2. We considered this threshold as significant as it is 50-fold higher than assigning each gene uniformly to all 222 gene clusters with equal degree of membership *μ *= 0.0045.

**Figure 8:**
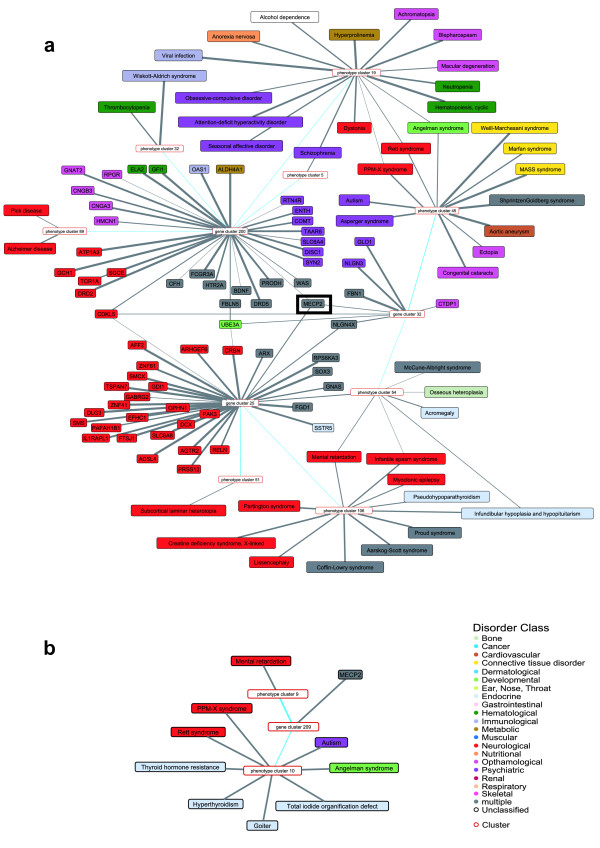
**The small-scale clustering in the neighborhood of MECP2**. We draw the results - the backbone network and the nodes' degrees of membership to clusters, thresholded by *μ *> 0.2 - of the small-scale clustering in the neighborhood of *MECP2 *using the fuzzy **(a) **and the hard clustering **(b)**. Nodes are colored according to their disorder class annotations. Blue edges indicate backbone interconnectivity, grey edges cluster assignment. Edge thickness indicates the degree of membership. *MECP2 *is connected to three gene clusters mainly covering neurological (red) and psychiatric (purple) genes. The seven interconnected disease clusters also represent mainly psychiatric and neurological disorders. Also unclassified disorders are present such as e.g. *Alcohol dependence *(white), which is classified as a mental and behavioral disorder. In a broader sense, however, it can be considered as psychiatric disorder. Applying the hard clustering **(b)**, *MECP2 *is assigned to gene cluster 209 which is connected to two disease clusters only. Although all associated disorders are identified correctly, in contrast to the fuzzy clustering no further information can be obtained from the decomposition.

As a showcase we chose *MECP2*, a protein that functions as a key factor in epigenetic transcriptional regulation. It is known to be involved in neurodevelopmental and psychiatric disorders such as *Autism*, *Mental retardation *and *Angelman syndrome *[3,27,28], and was assigned to three distinct gene clusters: 25 (*μ *= 0.42), 32 (*μ *= 0.31), 200 (*μ *= 0.24). These clusters mainly cover neurological (23%), psychiatric (81%) and pleiotropic (7%) genes having a degree of membership *μ *> 0.2. This is illustrated in Figure 8, where we visualized the backbone interconnectivity and the fuzzy clustering of the nodes in the neighborhood of *MECP2*.

We then analyzed the nine disease clusters interconnected with the three gene clusters in the backbone network. In total, 45 disorders representing mainly psychiatric (66%) and neurological (20%) disorders were assigned to eight disease clusters with a degree of membership of *μ *> 0.2; 6 out of 9 psychiatric disorders available in the network analyzed are present in three disease clusters.

Another large fraction of these disease clusters are disorders classified as *multiple*. Most of them (*Shprintzen-Goldberg syndrome *or *Aarskog Scott syndrome*) show also neurological diseases such as mental retardation [29,30]. We also identified the *ophthamological *disorder *Blepharospasm*, an adult-onset focal dystonia that causes involuntary blinking and eyelid spasms [31] for that a known polymorphism in the dopamine receptor *DRD5 *is associated with [32]. This is a subform of *Dystonia *and classified as a *neurological *disorder (ICD-10 G24.5) by the WHO [33].

Furthermore, we found *Anorexia nervosa *to be present in the analyzed clusters. It is annotated as a *nutritional *disorder by [3], however it represents a life-threatening complex psychiatric disorder [34]. Another so far unclassified disease *Alcohol dependence *was assigned to the interconnected cluster. It is classified as a mental and behavioral disorder (ICD-10 F10.2) and in a broader sense can be considered as psychiatric disorder.

In contrast, applying the hard clustering algorithm, *MECP2 *was assigned to a single gene cluster which is connected to two disease clusters. Although all associated disorders were identified correctly, no further information could be obtained from the clusters. However, [27] reports an epigenetic overlap in autism-spectrum neurodevelopmental disorders as *MECP2 *affects the regulation of *UBE3A *expression. These relations became immediately apparent in the cluster result of our fuzzy approach: Both genes were mutually assigned to gene cluster 25 that identifies the phenotypic and genotypic overlaps, whereas direct links to known connected genes are missing in the hard clustering (see Figure 8).

## Conclusions

The widespread application of high-throughput methods such as microarrays or next generation sequencing has considerably increased the amount of experimental data and the information available in biomedical literature that is accessible to text-mining approaches [35]. These data can usually be represented in terms of networks. Over the last years, networks have emerged as an invaluable tool for describing and analyzing complex systems. However, we need to take into account that network information is commonly available for various types of nodes. Especially integrative biological networks are *k*-partite [3,36].

Another important feature of biological networks is their hierarchical organization, implying that small groups of nodes organize in a hierarchical manner to increasingly larger groups on many different scales [24-26]. This necessitates the analysis of these objects on various resolution levels. Furthermore, many proteins or genes are pleiotropic, and often associated with many functions. Hence, clustering algorithms that assign elements into several functional modules are essential [10,12,37].

We presented a novel computationally efficient and scalable graph clustering algorithm that is capable to deal with all these described issues. Further, it does not require any *a priori *knowledge about the data set. Results on a tripartite network, constructed by integrating the human disease network and protein complexes, demonstrated that we could identify and interconnect biologically meaningful clusters on different scales. Overlapping modules gave a more comprehensive picture of e.g. gene-disease connections than looking at disjoint clusters alone. Summarizing, the proposed fuzzy clustering algorithm is suitable to compress and approximate the underlying topology of heterogeneous biological networks, which facilitates the understanding of such networks on multiple scales. It is freely available and readily applicable to many further problems.

## Methods

### Derivation of the update rules

We want to minimize *f*(*H*, *C*) in equation (1) using a local algorithm extending gradient descent. Let **D**^(*ij*)^: = **A**^(*ij*) ^- **C**^(*i*)^**B**^(*ij*)^(**C**^(*j*)^)^⊤ ^denote the residuals, then $f=\sum_{i<j,k,l}{\left(d_{kl}^{\left(ij\right)}\right)}^{2}$. Hence

$$\begin{array}{c} \frac{\partial f}{\partial b_{rs}^{\left(ij\right)}}=-2\sum_{kl}d_{kl}^{\left(ij\right)}c_{kr}^{\left(i\right)}c_{ls}^{\left(j\right)}\\ =-2{\left({\left(C^{\left(i\right)}\right)}^{⊤}D^{\left(ij\right)}C^{\left(j\right)}\right)}_{rs}\\ \frac{\partial f}{\partial c_{rs}^{\left(i\right)}}=-2\sum_{j>i,k,l}d_{rl}^{\left(ij\right)}b_{sk}^{\left(ij\right)}c_{lk}^{\left(j\right)}-2\sum_{j<i,kl}d_{kr}^{\left(ji\right)}c_{kl}^{\left(j\right)}b_{ls}^{\left(ji\right)}\\ =-2\sum_{j>i}{(D^{\left(ij\right)}C^{\left(j\right)}{\left(B^{\left(ij\right)}\right)}^{⊤})}_{rs}\\ \;-2\sum_{j<i}{\left({\left(D^{\left(ji\right)}\right)}^{⊤}C^{\left(j\right)}B^{\left(ji\right)}\right)}_{rs}. \end{array}$$

We assume an undirected *k*-partite graph, so **A**^(*ij*) ^is undefined for *i *>*j*. For simplicity of notation, we now set **A**^(*ij*)^: = (**A**^(*ji*)^)^⊤ ^for *i *>*j *(and similarly for the *k*-partite graph *H*). Then **D**^(*ij*) ^= (**D**^(*ji*)^)^⊤^, and the differential simplifies to

$$\frac{\partial f}{\partial c_{rs}^{\left(i\right)}}=-2\sum_{j\neq i}{\left(D^{\left(ij\right)}C^{\left(j\right)}{\left(B^{\left(ij\right)}\right)}^{⊤}\right)}_{rs}.$$

Altogether, by replacing the residuals, we have shown

$$\begin{array}{l} \frac{\partial f}{\partial b_{rs}^{\left(ij\right)}}=-2({\left(C^{\left(i\right)}\right)}^{⊤}A^{\left(ij\right)}C^{\left(j\right)}-\\ \;\;\;\;{{\left(C^{\left(i\right)}\right)}^{⊤}C^{\left(i\right)}B^{\left(ij\right)}{\left(\left(\right)\right)}^{⊤}C^{\left(j\right)})}_{rs}\\ \frac{\partial f}{\partial c_{rs}^{\left(i\right)}}=-2\sum_{j\neq i}(A^{\left(ij\right)}C^{\left(j\right)}{\left(B^{\left(ij\right)}\right)}^{⊤}-\\ \;\;\;\;{C^{\left(i\right)}B^{\left(ij\right)}{\left(C^{\left(j\right)}\right)}^{⊤}C^{\left(j\right)}{\left(B^{\left(ij\right)}\right)}^{⊤})}_{rs}. \end{array}$$

If we are to minimize *f *by alternating gradient descent, we start from an initial guess of **B**^(*ij*)^, **C**^(*i*)^. Then, we alternate between updates of the **B**^(*ij*) ^and the **C**^(*i*) ^with learning rates $\eta_{rs}^{\left(ij\right)}$ and $\eta_{rs}^{\left(i\right)}$, respectively:

$$\begin{array}{l} b_{rs}^{\left(ij\right)}\leftarrow b_{rs}^{\left(ij\right)}-\eta_{rs}^{\left(ij\right)}\frac{\partial f}{\partial b_{rs}^{\left(ij\right)}}\;\;\forall i,j:i<j\\ c_{rs}^{\left(i\right)}\leftarrow c_{rs}^{\left(i\right)}-\eta_{rs}^{\left(i\right)}\frac{\partial f}{\partial c_{rs}^{\left(i\right)}}\;\;\;\forall i \end{array}$$

These update rules have two disadvantages: first, the choice of update rate *η *(possibly different for **B**, **C **and *i*, *j*) is unclear; in particular, for too small *η *convergence may take too long or may not be achieved at all, whereas for too large *η *we may easily overshoot the minimum. Moreover, the resulting matrices may become negative. Hence we follow Lee and Seung's idea for NMF [13] and rewrite this into multiplicative update rules. We therefore choose update rates

$$\begin{array}{l} \eta_{rs}^{\left(ij\right)}:=\frac{b_{rs}^{\left(ij\right)}}{2{\left({\left(C^{\left(i\right)}\right)}^{⊤}C^{\left(i\right)}B^{\left(ij\right)}{\left(C^{\left(j\right)}\right)}^{⊤}C^{\left(j\right)}\right)}_{rs}}\;\text{and}\\ \eta_{rs}^{\left(i\right)}:=\frac{c_{rs}^{\left(i\right)}}{2{\left(\sum_{j\neq i}C^{\left(i\right)}B^{\left(ij\right)}{\left(C^{\left(j\right)}\right)}^{⊤}C^{\left(j\right)}{\left(B^{\left(ij\right)}\right)}^{⊤}\right)}_{rs}} \end{array}$$

Plugging this into the gradient descent equations, we finally get:

$$\begin{array}{l} b_{rs}^{\left(ij\right)}\leftarrow b_{rs}^{\left(ij\right)}\frac{{\left({\left(C^{\left(i\right)}\right)}^{⊤}A^{\left(ij\right)}C^{\left(j\right)}\right)}_{rs}}{{\left({\left(C^{\left(i\right)}\right)}^{⊤}C^{\left(i\right)}B^{\left(ij\right)}{\left(C^{\left(j\right)}\right)}^{⊤}C^{\left(j\right)}\right)}_{rs}}\\ c_{rs}^{\left(i\right)}\leftarrow c_{rs}^{\left(i\right)}\frac{{\left(\sum_{j\neq i}A^{\left(ij\right)}C^{\left(j\right)}{\left(B^{\left(ij\right)}\right)}^{⊤}\right)}_{rs}}{{\left(\sum_{j\neq i}C^{\left(i\right)}B^{\left(ij\right)}{\left(C^{\left(j\right)}\right)}^{⊤}C^{\left(j\right)}{\left(B^{\left(ij\right)}\right)}^{⊤}\right)}_{rs}} \end{array}$$

Commonly, in order to extend cost functions in (unipartite) data clustering to include fuzzy clusters, a so-called *fuzzification factor *is introduced [11,38]]. Instead of squared norm minimization of the residuals **D**^(*ij*)^, a higher residual power is minimized, which results in overlapping non-trivial cluster assignments. However, we see that in our examples, already the standard case is sufficient. This is because we are interested in co-clustering, which is different from standard data clustering where only a unipartite graph and hence **C**^(*i*) ^= **C**^(1) ^is assumed.

### Evaluation on simulated data

We built a random, modularly structured *k*-partite network as follows: We fix the number of clusters *m_i _*of nodes with color *i*, *i *= 1, ... *k*. The backbone graph is initialized by *m_i _*× *m_j_*-matrices **B**^(*ij*) ^filled with zeros. We added uniformly random ones in each column according to a set percentage *α *(here on average *α *≥1 ones in each column) such that each row has at least a single non-zero entry. In order to construct the actual network **A**, we split up **A**^(*ij*) ^into *m_i _*· *m_j _*blocks of a fixed chosen clustersize (here 10). We fixed a cluster connectivity *β *and a random connectivity *γ *<*β*. Now, for each non-zero entry in **B**^(*ij*)^, we set the corresponding block of **A**^(*ij*) ^to a random Erdös-Rényi graph [39] with density *β*. Finally the clusters are connected by replacing each zero block of **A**^(*ij*) ^with an Erdös-Rényi graph of the lower connectivity *γ*. We analyzed 1000 realizations of four network prototypes with increasing complexity (parameters are given in Table 1). In order to compare algorithm performance, we determined algorithm runtime, final cost function value and quality of cluster estimation. Cluster estimation quality was measured by the summed up Frobenius norms of the difference between the true **C**^(*i*) ^and the estimated ${\hat{C}}^{\left(i\right)}$, where clusters have been permuted such as to give minimal difference (permutation indeterminacy).

**Table 1:** Random data models for evaluation of the fuzzy clustering algorithm

model	*k*	**m**	*α*	*β*	*γ*	description
1	2	(3, 3)	1	0.7	0.2	equal-sized, no overlap
2	2	(3, 4)	1	0.7	0.2	no cluster overlap
3	3	(3, 4, 5)	1.2	0.6	0.1	3-partite, low-noise
4	3	(3, 4, 5)	1.2	0.8	0.2	3-partite, noisy

Parameters for the simulated data models. *k *denotes the number of partitions of the network, **m **is a vector with the number of clusters in each partition, *α *the backbone connectivity, *β *the cluster and *γ *the noise connectivity.

### Construction of a disease-gene-protein complex graph

We constructed a layered, tripartite graph by enlarging the human disease network [3] by all human protein complexes from the CORUM core set (as of July 2009) [16]. Integrating both data sets resulted in a graph of 5672 nodes and 7795 edges with all genetic disorders, all known disease genes and human protein complexes. We extracted the largest connected component resulting in a network with |*V*| = 3737 and |*E*| = 6219. It consists of 854 complexes (*V_c_*), 590 diseases (*V_d_*) and 2293 genes (*V_g_*) (see Additional File 7).

### Parameter determination

We determined parameters for clustering on different scales. For large-scale clustering, we approximated the number of clusters to be found for each node type by limiting the maximal number of gene clusters *m_g _*for *V_g _*to *m_g_*=$m_{g}=\left\lfloor \sqrt{|V_{g}|/2}\right\rfloor$ as suggested in [40]. The number of complex clusters *m_c _*for *V_c _*and disease clusters *m_d _*for *V_d _*were then scaled according to *m_g _*by *_i _*= $m_{i}=\left\lceil m_{g}\sqrt{\left|V_{i}|/|V_{g}\right|}\right\rceil$, where *i *∈ {*c*, *d*}. To detect smaller clusters, we set the maximum number of gene clusters to *m_g _*for *V_g _*according to *m_g _*= $m_{g}=\left\lceil \frac{\left|V_{g}\right|}{10}\right\rceil$. This resulted in a minimum average cluster size of 10 genes. Parameters *m_c _*and *m_d _*for *V_c _*and *V_d _*were scaled as previously.

### Cluster evaluation

We validated the gene clusters using Gene Ontology (GO) enrichment analysis. To this end, the genes used in the analysis (degree of membership *μ *> 0.2) were tagged with their respective GO categories and analyzed within each cluster for overrepresentation of certain categories versus the ”background” level of the population (in this case, all genes in the tripartite graph). We used Ontologizer [41] with the setting ”Parent-Child-Intersection” restricting the analysis to the *biological process *category. For multiple testing correction we employed Bonferroni correction. To assign GO terms to gene sets, a *p*-value cutoff of 0.05 was used.

For evaluating the cluster interconnectivity we employed FunCat [42] classifications for all genes and protein complexes. We used FunCat, as Gene Ontology associations for genes could be mapped to their according FunCat categories, but not vice versa. A subset of 13 main categories was used, subcategory annotations were mapped to corresponding main category terms. Disorder classifications for genes and diseases were taken from [3], where classification classes *grey *and *multiple *were combined for pleiotropic genes (see Additional File 8). We calculated Pearson's correlation coefficients between cluster FunCat/disorder annotations by weighting a cluster element's classification by its degree of membership to the particular cluster. The difference score between normalized backbone interconnectivity and annotation correlation was determined using the Frobenius norm of their difference.

### Null model

Null models for the evaluation of the backbone graph in the large-scale clustering were generated by applying a weighted bipartite randomization procedure to each partition-cluster subgraph **C**^(*i*)^. To this end, we generalized the degree preserving rewiring of complex networks first introduced by [43]. In the weighted case, one has to decide between preserving either the number of neighbors of all nodes, or the total weight of their adjacent edges. We chose to maintain the first quantity: In every randomization step we randomly picked two edges and exchange their endpoints of the partition type, thereby keeping the weights attached to the edges. With this we also conserved the weighted degree of the partition nodes which reflects the right-stochasticity of the fuzzy clusterings. The degree of randomization can be monitored by a loss of degree-correlations between first and second neighbors. In practice, correlations vanish after about one randomization step per edge. So, for our analyses we used five times this number as suggested in [44]. The *p*-values were calculated over 10000 runs.

## Availability and requirements

**Project name: **Fuzzy clustering of k-partite graphs.

**Project home page: **http://cmb.helmholtz-muenchen.de/fuzzyclustering.

**Operating system(s): **Platform independent.

**Programming language: **MATLAB/Octave.

**Other requirements: **MATLAB 7.1 or higher (no additional toolboxes required) or Octave.

**License: **Free for non-commercial purposes.

## Authors' contributions

The first two authors, MLH and FB, should be regarded as joint first authors. Conceived and designed the study: MLH, FB, VS, FJT. Performed the experiments: MLH, FB. Analyzed the data: MLH. Developed methods/analysis tools: MLH, FB, FJT. Wrote the paper: MLH, FB, FJT. All authors read and approved the final version of the manuscript.

## Supplementary Material

Additional file 1 **MATLAB source code**. Fuzzy k-partite graph clustering algorithm for MATLAB. Click here for file

Additional file 2 **Octave source code**. Fuzzy k-partite graph clustering algorithm for Octave. Click here for file

Additional file 3 **Simulations on algorithm runtime**. Verification of the estimation of the algorithm's time complexity by simulations. Click here for file

Additional file 4 **Simulation on cluster stability**. Analysis of the algorithm's stability towards the random initialization. Click here for file

Additional file 5 **The chosen number of clusters**. Analysis of the cost function as an indicator for determining the number of clusters. We study the stability of the clusterings with respect to this choice and give evidence that the gene-disease-complex graph is modularly structured. Click here for file

Additional file 6 **GO enrichment analysis for the gene clusters from the large-scale clustering**. Tables 1-10 show the GO (Gene Ontology) enrichment using Ontologizer [41] for the ten gene clusters in the large-scale clustering. We used only genes having a degree of membership μ > 0.2 (see Methods). Click here for file

Additional file 7 **Integrated tripartite network**. Illustration of the largest connected component of the layered, tripartite graph gene-disease-protein complex network. It consists of 2293 gene (green), 590 disease (red) and 854 complex (blue) nodes connected by 6219 edges. Click here for file

Additional 8 **FunCat and disorder class annotation tables**. Table 1 shows the FunCat classes used for evaluating the gene and protein complex clusters. A subset of 13 FunCat main categories was taken from CORUM. Table 2 represents the 20 primary disorder classes retrieved from Goh et al. (2007). Additional classes are *multiple, grey and unclassfied*. Click here for file
