# Supplementary material for: Structuring heterogeneous biological information using fuzzy clustering of k-partite graphs
Source: BMC Bioinformatics. 2010 Oct 20;11:522. doi: 10.1186/1471-2105-11-522 (PMC3247861; doi:10.1186/1471-2105-11-522)
Supplement: Additional file 3 — Simulations on algorithm runtime. Verification of the estimation of the algorithm's time complexity by simulations. [file 1471-2105-11-522-S3.PDF]

Additional file 3 —Simulations on algorithm runtime.

In order to verify the estimation of the algorithm's time complexity in the main text, we performed three simulations. In Figure 1, we demonstrate the *linear* dependency of the algorithm's runtime on the partition size n_i of any partition i. Figure 2 confirms the quadratic dependency of the runtime on the partition size in graphs with equally sized partitions. Runtimes were measured with an Intel(R) Xeon CPU with 2.00GHz and the MATLAB implementation in Additional file 3.

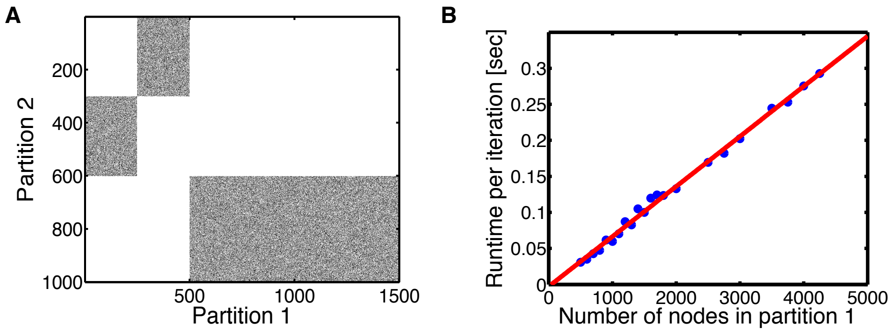

Figure 1: To analyze algorithm runtime, we generated bipartite networks consisting of three unconnected components: we chose to have three hard clusters in each partition, each of them connected to only one cluster of the other partition. Two nodes of different color stemming from linked clusters were connected with a probability of 0.4. **(A)** illustrates the adjacency matrix of one sample (with 1500 nodes in partition 1) as heat-map, where white denotes zeros and black denotes ones. We varied the size of partition 1 between 500 and 4500 nodes, while partition 2 always contained 1000 nodes. Thereby, we kept the number of clusters in the graph and also the number of clusters extracted by the algorithm constant. We measured the average runtime for a single iteration when decomposing these graphs. This runtime is plotted in **(B)**. The simulation clearly confirms the *linear* dependency of our algorithm's runtime on the partition size n_i of a partition i, $i = 1 \ldots k$.

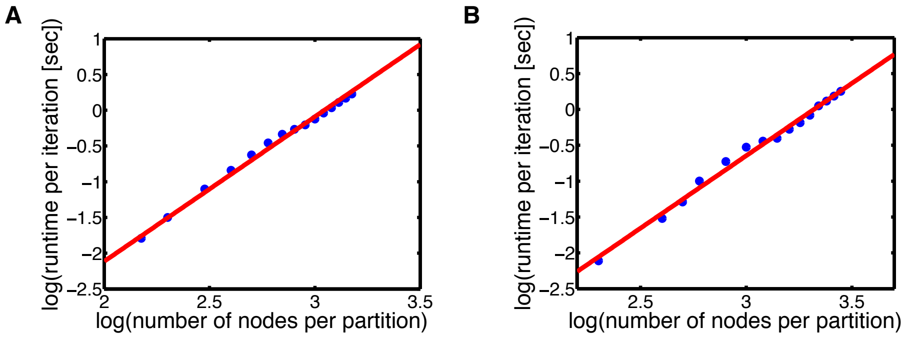

Figure 2: **(A)** further confirms the theoretical analysis of the algorithm's time complexity in the main text. Again, we simulated bipartite graphs with three hard clusters per partition that consisted of three unconnected components. Now, we varied the sizes of both partitions, which are always equally large. Blue dots indicate the observed runtimes, the red line is a linear fit with a slope of 2.03. **(B)** shows the dependency of the runtime on the product of the two largest partition sizes in a tripartite example with equally sized partitions. These graphs were constructed analogously to the bipartite ones. Blue dots indicate the observed runtimes, the red line is a linear fit with a slope of 2.01. Thus, we verified the quadratic dependency of our algorithm on the partition size in the case of equally sized partitions.
