# Supplementary material for: Structuring heterogeneous biological information using fuzzy clustering of k-partite graphs
Source: BMC Bioinformatics. 2010 Oct 20;11:522. doi: 10.1186/1471-2105-11-522 (PMC3247861; doi:10.1186/1471-2105-11-522)
Supplement: Additional file 4 — Simulation on cluster stability. Analysis of the algorithm's stability towards the random initialization. [file 1471-2105-11-522-S4.PDF]

Additional file 4 —Simulations on algorithm stability.

In cluster analysis, the validity of specific solutions represents an important criterium to determine the performance of clustering algorithms. The reproducibility and therefore stability of clustering results has to be analyzed since different outcomes may have significant impacts on the interpretation of the results. It is widely known that NMF-based methods are sensitive to initializations and therefore may converge to different solutions due to different (random) initializations. In order to identify stable results multiple restarts are generally used [1, 2].

To evaluate the reproducibility of our NMF-based local algorithm we compared the clustering results of a bipartite network with well defined cluster structure using multiple restarts. To this end, we generated a bipartite network consisting of three unconnected components: we chose to have three hard clusters ($k = 3$) in each partition, each of them connected to only one cluster of the other partition. Two nodes of different color stemming from linked clusters were connected with a probability of 0.4. The adjacency matrix (with 900 nodes in partition 1 and 800 nodes in partition 2) is illustrated in Figure 1.
To determine how stable the clustering results are, we applied our algorithm to this bipartite network 100 times decomposing both partitions into $k = 3$ clusters each. In order to compare the clustering results and to quantify their stability an appropriate similarity measure (stability score) has to be used. Garge et al. [3] proposed to use Cramer's v^2 as a measure for the degree of replicability. Cramer's v^2 quantifies the degree of association in contingency tables larger than 2×2 reflecting the frequency distribution of data classification (cluster assignment) by two systems simultaneously. However, crisp assignment of data points to clusters – every data point is assigned to the cluster showing maximum degree of membership – is required. As the process of crisp assignment may affect the stability score and an important feature of our proposed method is the detection of overlapping clusters, we use a different similarity measure, the so-called Fuzzy Rand Index (FRI) recently proposed by Hüllermeier et al. [4]. The FRI allows to compare fuzzy partitions and therefore to determine how close the fuzzy memberships obtained in different runs are (see equations (1,2)). It ranges from 0 to 1, with 0 indicating no relationship and 1 indicating a perfect reproducibility.

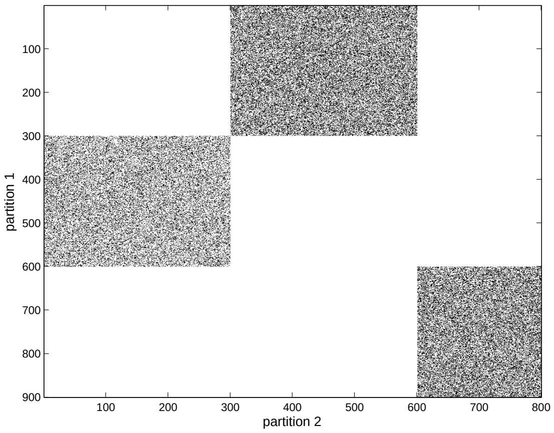

Figure 1: The adjacency matrix (with 900 nodes in partition 1 and 800 nodes in partition 2) used for the cluster stability analysis is illustrated as heat-map, where white denotes zeros and black denotes ones.

As defined in the manuscript $C := (\mathbf{C}^{(i)})_{i=1,\dots,k}$ represents a fuzzy clustering of the graph partition the graph, with $\mathbf{C}^{(i)}$ being the matrix of the degrees of membership in partition i. A fuzzy equivalence relation on $\mathbf{C}^{(i)}$ is defined in terms of a similarity measure on the associated degrees of membership vectors. Generally, this relation can be defined via any distance measure on $[0,1]^{m_i}$ that yields values in $[0,1]$. We chose to employ the maximum norm and define the distance $D_{\mathbf{C}^{(i)}}(r,s)$ between the degrees of memberships of two nodes $r, s \in V_i$

$$D_{\mathbf{C}^{(i)}}(r,s) := \max_t \left| \mathbf{C}^{(i)}_{st} - \mathbf{C}^{(i)}_{rt} \right| . \tag{1}$$

In other words, the distance between two fuzzy memberships $\mathbf{C}^{(i)}_{st}$ and $\mathbf{C}^{(i)}_{rt}$ is the largest of their differences along any coordinate dimension.
Now, given the fuzzy cluster assignments $\mathbf{C}^{(i)}$ and $\mathbf{C}^{(i)\prime}$ resulting from two different initializations we calculate the FRI that is defined as the degree of concordance.

$$FRI(\mathbf{C}^{(i)}, \mathbf{C}^{(i)\prime}) = 1 - \frac{\sum_{r<s} |D_{\mathbf{C}^{(i)}}(r,s) - D_{\mathbf{C}^{(i)\prime}}(r,s)|}{n_i\,(n_i - 1)\,/2} \tag{2}$$

where n_i is the number of nodes in partition i. In our case, $n_1 = 900$ for partition 1 and $n_2 = 800$ for partition 2. For a detailed analysis of this measure we refer to [4].

In our example, we calculated the pairwise similarity of all clustering results to the reference fuzzy memberships for both partitions separately. The resulting FRI distribution is shown in Figure 2.

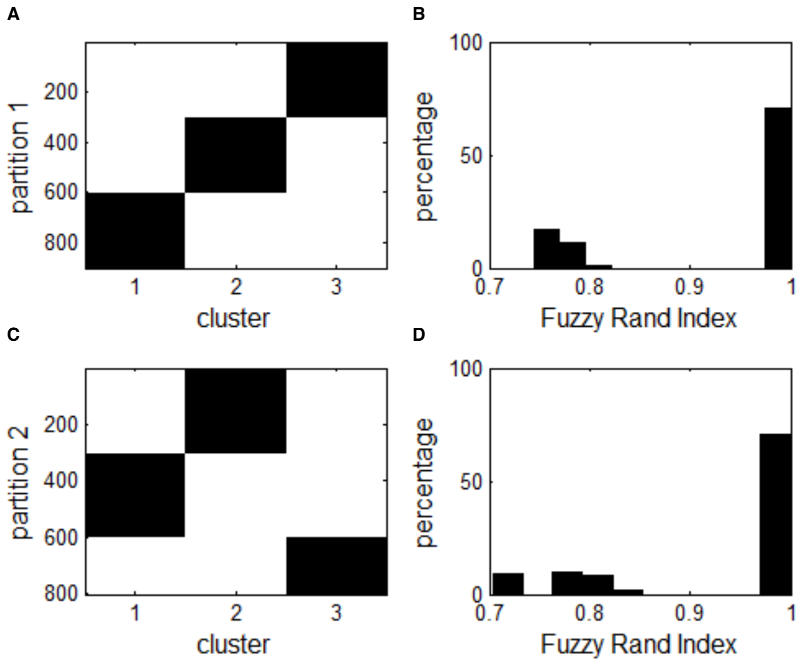

Figure 2: **(A)** Good decomposition of partition 1 into the true cluster structure, illustrated as heat-map, where white denotes zeros and black denotes ones. We compared the clustering results of 100 runs with the extracted cluster structure to evaluate the reproducibility of our algorithm's outcome. The FRI was used to quantify the stability of the clustering results. **(B)** Distribution of the FRI over 100 runs. We reach a FRI of 1 in more than 70 percent, indicating a perfect reproducibility. **(C)** Partition 2 is well decomposed into its predefined cluster structure with 3 clusters. Again, in more than 70 percent a perfect reproducibility score with FRI of 1 is reached. The FRI distribution of the 100 comparisons of partition 2 is shown in **(D)**.

In more than 70 percent, we reach a FRI of 1, indicating that our algorithm produces stable results with close fuzzy memberships. However, although the input graph has a well defined cluster structure, our algorithm does not always converge to a meaningful decomposition. Sometimes, only one cluster is determined correctly (see also Figure 3). This is due to the fact that our NMF-based algorithm employs a local optimization strategy and may therefore converge to different local minima due to random initializations.

A

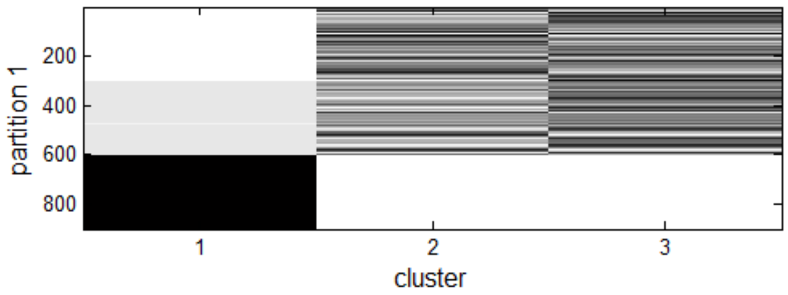

B

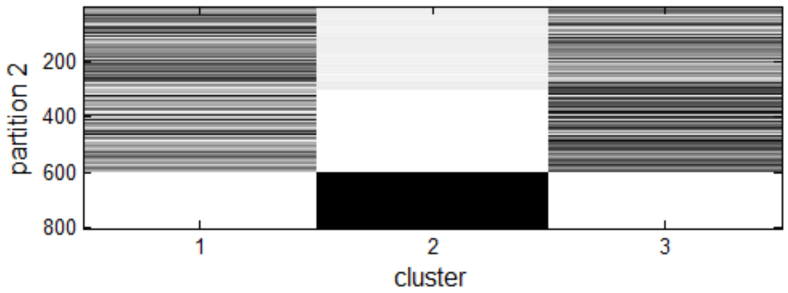

Figure 3: **(A)** Our algorithm does not always decompose partition 1 into the predefined cluster structure with 3 clusters. Moreover, it results in one hard cluster and two strongly overlapping clusters. **(B)** Partition 2 is also not always decomposed clearly. Due to random initialization the NMF based algorithm may converge to a local minimum and therefore not decompose the network into the real cluster structure correctly.

References

[1] Langville AN, Meyer CD, Albright R: **Initializations for the Nonnegative Matrix Factorization**. *KDD 2006 Philadelphia, PA USA* 2006.

[2] Devarajan K: **Nonnegative matrix factorization: an analytical and interpretive tool in computational biology.** *PLoS Comput Biol* 2008, **4**(7):e1000029.

[3] Garge NR, Page GP, Sprague AP, Gorman BS, Allison DB: **Reproducible clusters from microarray research: whither?** *BMC Bioinformatics* 2005, **6 Suppl 2**:S10.

[4] Hüllermeier E, Rifqi M: **A Fuzzy Variant of the Rand Index for Comparing Clustering Structures**. In *IFSA/EUSFLAT Conf.*. Edited by Carvalho JP, Dubois D, Kaymak U, da Costa Sousa JM 2009:1294–1298.
