# Supplementary material for: Structuring heterogeneous biological information using fuzzy clustering of k-partite graphs
Source: BMC Bioinformatics. 2010 Oct 20;11:522. doi: 10.1186/1471-2105-11-522 (PMC3247861; doi:10.1186/1471-2105-11-522)
Supplement: Additional file 5 — The chosen number of clusters. Analysis of the cost function as an indicator for determining the number of clusters. We study the stability of the clusterings with respect to this choice and give evidence that the gene-disease-complex graph is modularly structured. [file 1471-2105-11-522-S5.PDF]

Additional file 5 —The chosen number of clusters.

In this Additional file, we illustrate the observed behavior of the cost-function in Equation (1) of the main text with respect to variations in the numbers of extracted clusters m_k. Moreover, we study the stability of graph decompositions with respect to this number in a toy example. We demonstrate that the degree of membership matrices obtained from graphs with clear cluster structure are quite different from those of graphs lacking this property. Analyzing the clusters found in the gene-disease-protein complex graph we give evidence that this graph indeed is modularly structured at the desired resolution level.

Heuristic determination of m_k

Again, we note that our cost function is in theory monotonous in the number of clusters m_k. The determination of these numbers (one for each node type) is difficult. Even in the case of unipartite k-means or Principal Component Analysis there is no direct and computationally simple solution. In the k-partite setting with $k > 2$ a brute-force sampling of the parameter space is still out of reach. We therefore employ an – admittedly crude – heuristics. We plot the cost function depending on the cluster number – not the true value, but the estimated value for the decomposition. Then we look for drops in the cost function, or plateaus. In Figure 1 we validate this in a toy example where we see steep drops between two and six clusters detected, followed by a flat plateau.

The cluster structure depending on m

Using the first example from Figure 1, a bipartite graph with six well defined, yet not hard clusters, we shortly study the dependency of the clusters on the cluster number m, see Figure 2. In this example we see that extracting less clusters than really present, our algorithm identifies clusters composed by the union of true ones. If we want to extract more clusters than the correct number, it splits up true clusters into strongly overlapping sub-clusters. Hence, even when not extracting the exactly correct number of clusters, the decomposition obtained is meaningful and allows for a deeper interpretation of the extracted clusters.

Histograms of the degrees of membership

Histograms of the obtained degrees of membership show a characteristic behavior: if the graph contains some well defined cluster structure, they have an U-like shape: we find a large number of high degrees of membership almost up to one, see Figure 2. In graphs without cluster structure, compare Figure 3, these large values are completely missing. Hence, such histograms can be employed to give an indication whether a graph is modularly organized or not.
Figure 4 shows the degrees of membership from the large-scale clustering of the gene-disease-protein complex graph. In all partitions we find a large number of high degrees of membership. This gives evidence for the presence of a well defined cluster structure in the studied real-world application. The number of medium degrees of memberships shows the need for a fuzzy approach, especially in the case of the disease partition.

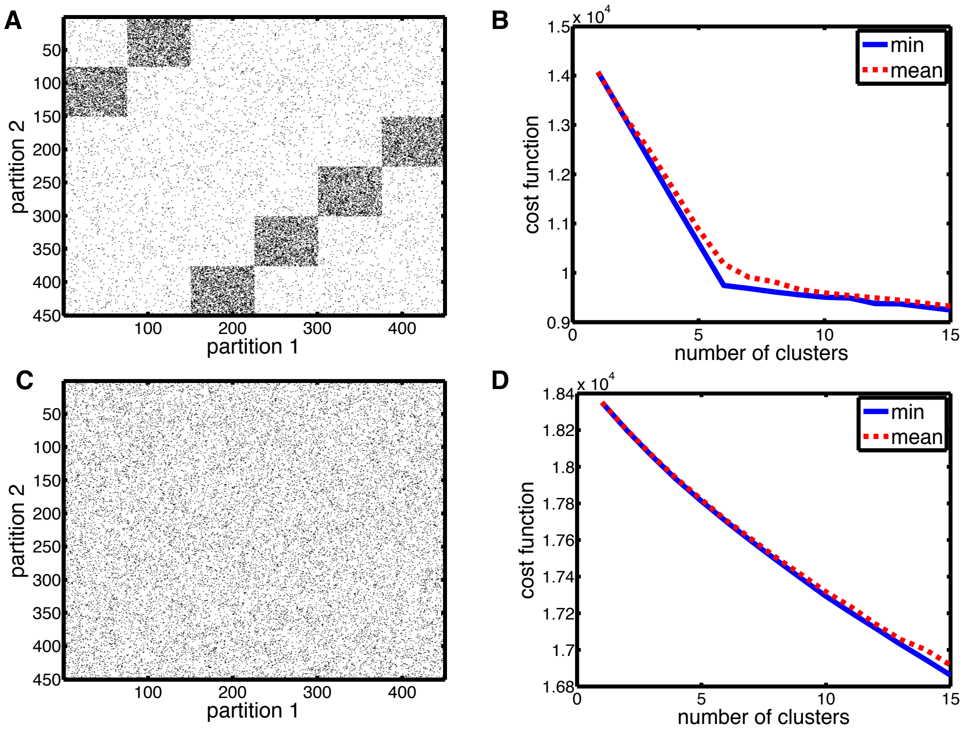

Figure 1: To analyze the profile of the cost function, we generated two bipartite example graphs. Their adjacency matrices are illustrated in **(A)** and **(C)** as heatmaps (black codes for ones, white for zeros). The first graph has six obvious (yet not hard) clusters in each partition, each of them connected to only one cluster of the other partition. It contains 75 nodes per cluster and two nodes of different color stemming from linked clusters are connected with a probability of 0.4. Additionally, we introduced random connections between the other nodes with a probability of 0.05. The second graph also contains 450 nodes per partition, but has no cluster structure (all pairs of nodes connected with probability 0.12).
(B) and **(D)** show the profile of the cost function after algorithm convergence (average and minimum value over 25 runs) when extracting between two and 15 clusters. While in the second, cluster-free example there is no structure in the profile of the cost function, the well defined cluster structure of the first example has a sharp break. For $m = 1 \dots 6$, i.e. until all present six clusters detected, we observe steep drops in the cost function, followed by a flat plateau with little refinement for $m > 6$.

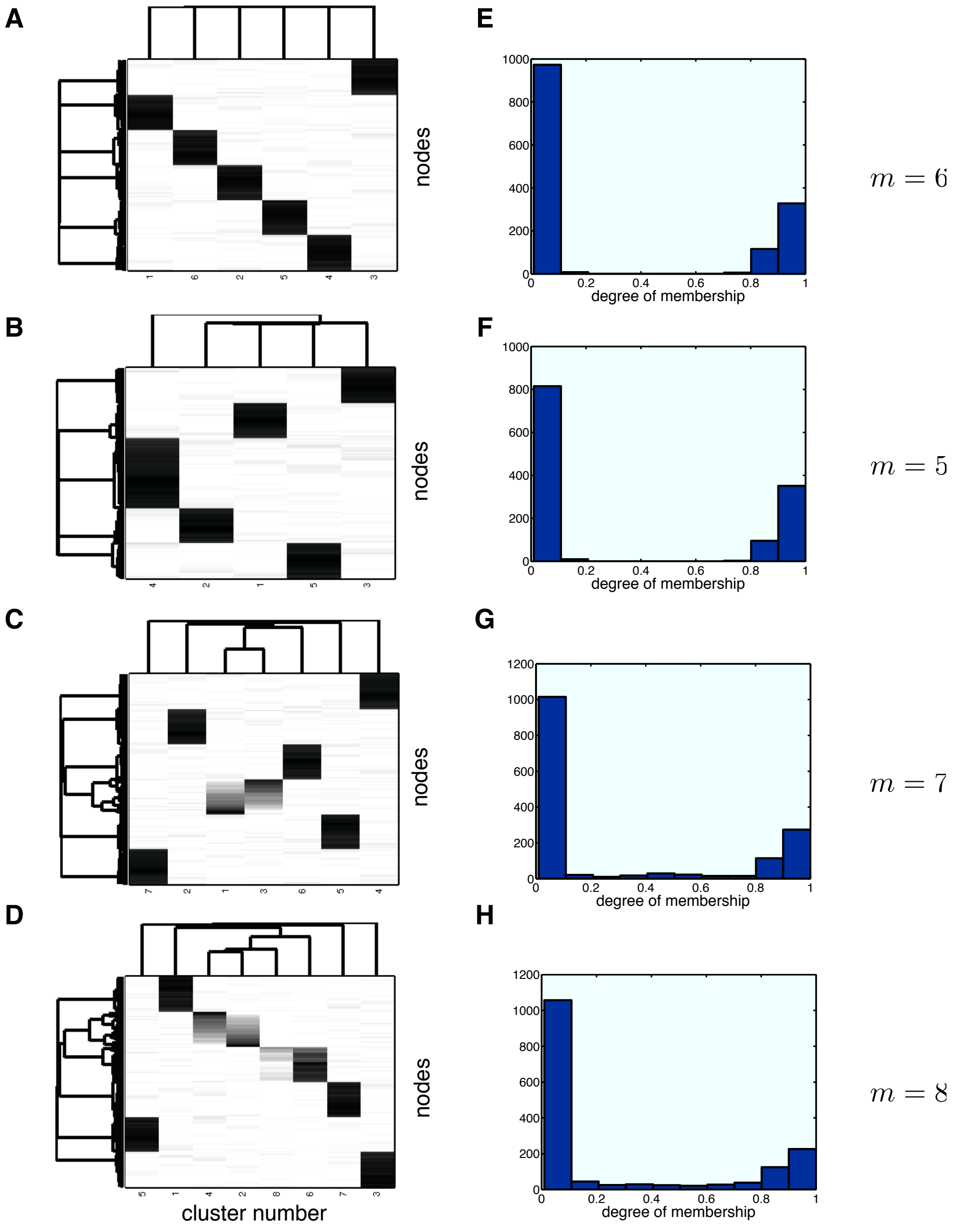

Figure 2: **(A)** to **(D)** give hierarchical clusterings of the degree of membership matrices obtained for the true $m = 6$ and also for $m = 5$ and $m = 7, 8$. In each case, we show the best of 25 runs. The algorithm shows a nice behavior: If we extract only five clusters, it detects four of the six clusters, the fifth cluster being the union of the last two true clusters. If we extract more than six clusters, it splits up true clusters into two strongly overlapping sub-clusters. In **(E)** to **(H)** we give histograms of the degrees of membership obtained in these situations (for better recognizability, we counted only entries ≥ 0.01). These histograms show a typical U-like shape, with a peak at small entries and a second peak at large degrees of membership around one indicating the well defined cluster structure of the studied graph.

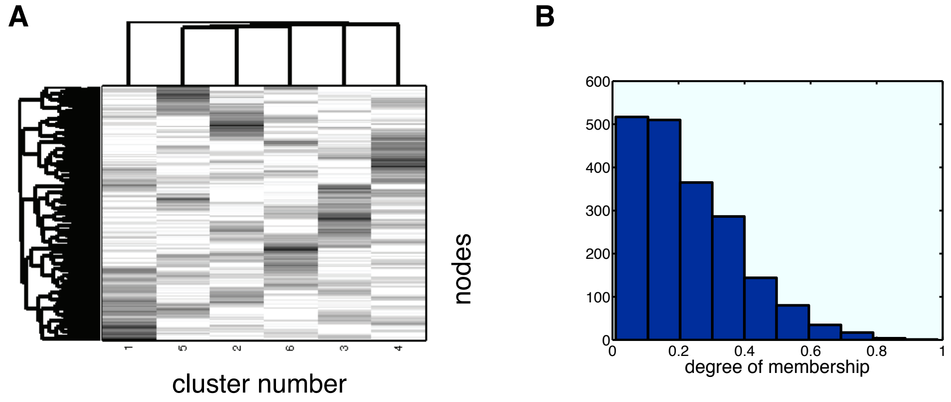

Figure 3: **(A)** gives a hierarchical clustering of the best of 25 decompositions of the graph from Figure 1 **(C)**, which has no cluster structure. Although the algorithm identifies clusters visible in this illustration, they are not well defined and blurred out. Consequently, in the histogram of the degrees of membership we see a large number of small values, the peak at degrees of membership above 0.8 is missing.

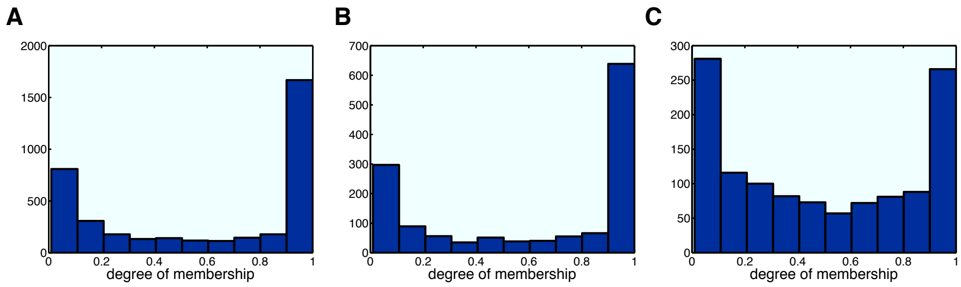

Figure 4: The degrees of membership from the large-scale clustering of the gene-disease-protein complex graph: **(A)** genes, **(B)** protein complexes, **(C)** diseases. The U-like shape gives evidence for the existence of a well defined cluster structure. Again, for better recognizability, we counted only entries ≥ 0.01.
