# Supplementary material for: Structuring heterogeneous biological information using fuzzy clustering of k-partite graphs
Source: BMC Bioinformatics. 2010 Oct 20;11:522. doi: 10.1186/1471-2105-11-522 (PMC3247861; doi:10.1186/1471-2105-11-522)
Supplement: Additional file 6 — GO enrichment analysis for the gene clusters from the large-scale clustering. Tables 1-10 show the GO (Gene Ontology) enrichment using Ontologizer [41] for the ten gene clusters in the large-scale clustering. We used only genes having a degree of membership μ > 0.2 (see Methods). [file 1471-2105-11-522-S6.PDF]

Tables 1-10 show the GO (Gene Ontology) enrichment using Ontologizer [1] for the ten gene clusters in the rough clustering. We used only genes having a degree of membership $\mu > 0.2$ and the setting "Parent-Child-Intersection" restricting the analysis to the *biological process* category. To assign GO terms to gene sets, Bonferroni correction with a p-value cutoff of 0.05 was used for multiple testing correction.

Tables

Table 1 - Gene cluster 1 - GO enrichment analysis using Ontologizer [1] with settings "Parent-Child-Intersection/Bonferroni". For this analysis, a total of 2179 genes were in the population set, of which a total of 273 genes were in the study set.

ID	Name	p-Value	p-Value (Adj)	Study Count	Population Count
GO:0006259	DNA metabolic process	2.042×10^{-26}	3.075×10^{-23}	99	226
GO:0051716	cellular response to stimulus	1.334×10^{-16}	2.009×10^{-13}	93	324
GO:0006950	response to stress	4.663×10^{-13}	7.023×10^{-10}	105	466
GO:0071103	DNA conformation change	4.595×10^{-12}	6.919×10^{-9}	25	46
GO:0065004	protein-DNA complex assembly	9.804×10^{-12}	1.476×10^{-8}	19	29
GO:0007049	cell cycle	2.100×10^{-11}	3.162×10^{-8}	79	321
GO:0006996	organelle organization	4.274×10^{-11}	6.436×10^{-8}	93	405
GO:0006807	nitrogen compound metabolic process	2.109×10^{-9}	3.176×10^{-6}	179	1054
GO:0060249	anatomical structure homeostasis	6.764×10^{-9}	1.019×10^{-5}	19	47
GO:0006260	DNA replication	6.575×10^{-8}	9.903×10^{-5}	56	95
GO:0006974	response to DNA damage stimulus	8.615×10^{-8}	1.297×10^{-4}	83	185
GO:0043170	macromolecule metabolic process	3.931×10^{-7}	5.921×10^{-4}	208	1392
GO:0051276	chromosome organization	2.983×10^{-6}	0.00449	71	226
GO:0032845	negative regulation of homeostatic process	1.153×10^{-5}	0.01737	10	15
GO:0032200	telomere organization	1.209×10^{-5}	0.01821	17	23
GO:0033036	macromolecule localization	1.693×10^{-5}	0.02550	26	215
GO:0006297	nucleotide-excision repair, DNA gap filling	2.194×10^{-5}	0.03303	16	17

Table 2 - Gene cluster 2 - GO enrichment analysis using Ontologizer [1] with settings "Parent-Child-Intersection/Bonferroni". For this analysis, a total of 2179 genes were in the population set, of which a total of 137 genes were in the study set.

ID	Name	p-Value	p-Value (Adj)	Study Count	Population Count
GO:0051340	regulation of ligase activity	3.005×10^{-23}	2.560×10^{-20}	37	59
GO:0070647	protein modification by small protein conjugation or removal	3.691×10^{-22}	3.145×10^{-19}	42	119
GO:0006508	proteolysis	4.254×10^{-19}	3.625×10^{-16}	42	171
GO:0070271	protein complex biogenesis	2.716×10^{-12}	2.314×10^{-9}	43	195
GO:0043933	macromolecular complex subunit organization	3.147×10^{-12}	2.681×10^{-9}	43	287
GO:0009056	catabolic process	5.315×10^{-10}	4.528×10^{-7}	45	251
GO:0007049	cell cycle	6.420×10^{-10}	5.470×10^{-7}	49	321
GO:0006354	RNA elongation	8.057×10^{-10}	6.865×10^{-7}	21	44
GO:0044092	negative regulation of molecular function	9.132×10^{-9}	7.780×10^{-6}	38	136
GO:0043632	modification-dependent macromolecule catabolic process	3.625×10^{-8}	3.089×10^{-5}	39	92
GO:0010498	proteasomal protein catabolic process	6.004×10^{-8}	5.115×10^{-5}	38	71
GO:0065007	biological regulation	6.984×10^{-7}	5.951×10^{-4}	110	1343
GO:0031145	anaphase-promoting complex-dependent proteasomal ubiquitin-dependent protein catabolic process	1.460×10^{-6}	0.00124	37	53
GO:0051246	regulation of protein metabolic process	2.760×10^{-6}	0.00235	42	208
GO:0065009	regulation of molecular function	3.297×10^{-6}	0.00281	48	329
GO:0044093	positive regulation of molecular function	4.161×10^{-6}	0.00355	46	233
GO:0019222	regulation of metabolic process	5.002×10^{-6}	0.00426	98	858
GO:0030522	intracellular receptor-mediated signaling pathway	9.187×10^{-6}	0.00783	11	39
GO:0044085	cellular component biogenesis	9.747×10^{-6}	0.00830	45	390
GO:0022402	cell cycle process	3.190×10^{-5}	0.02718	46	229

Table 3 - Gene cluster 3 - GO enrichment analysis using Ontologizer [1] with settings "Parent-Child-Intersection/Bonferroni". For this analysis, a total of 2179 genes were in the population set, of which a total of 125 genes were in the study set.

ID	Name	p-Value	p-Value (Adj)	Study Count	Population Count
GO:0007049	cell cycle	6.935×10^{-6}	0.00696	38	321
GO:0051716	cellular response to stimulus	7.358×10^{-6}	0.00738	33	324

Table 4 - Gene cluster 4 - GO enrichment analysis using Ontologizer [1] with settings "Parent-Child-Intersection/Bonferroni". For this analysis, a total of 2179 genes were in the population set, of which a total of 100 genes were in the study set.

ID	Name	p-Value	p-Value (Adj)	Study Count	Population Count
GO:0016265	death	5.938×10^{-10}	8.551×10^{-7}	41	343
GO:0006793	phosphorus metabolic process	6.373×10^{-10}	9.178×10^{-7}	37	270
GO:0008283	cell proliferation	7.698×10^{-10}	1.109×10^{-6}	38	294
GO:0032502	developmental process	3.721×10^{-8}	5.358×10^{-5}	61	750
GO:0023052	signaling	1.511×10^{-7}	2.176×10^{-4}	55	657
GO:0007154	cell communication	3.287×10^{-7}	4.734×10^{-4}	35	316
GO:0043412	macromolecule modification	4.942×10^{-7}	7.116×10^{-4}	40	399
GO:0050896	response to stimulus	1.157×10^{-6}	0.00167	59	773
GO:0032501	multicellular organismal process	3.528×10^{-6}	0.00508	64	900

Table 5 - Gene cluster 5 - GO enrichment analysis using Ontologizer [1] with settings "Parent-Child-Intersection/Bonferroni". For this analysis, a total of 2179 genes were in the population set, of which a total of 299 genes were in the study set.

ID	Name	p-Value	p-Value (Adj)	Study Count	Population Count
GO:0022610	biological adhesion	1.050×10^{-14}	1.678×10^{-11}	59	158
GO:0016043	cellular component organization	1.254×10^{-10}	2.003×10^{-7}	158	777
GO:0070979	protein K11-linked ubiquitination	2.813×10^{-8}	4.495×10^{-5}	9	9
GO:0007229	integrin-mediated signaling pathway	2.926×10^{-8}	4.675×10^{-5}	24	34
GO:0032774	RNA biosynthetic process	5.243×10^{-8}	8.378×10^{-5}	122	437
GO:0009058	biosynthetic process	2.206×10^{-7}	3.525×10^{-4}	164	915
GO:0019222	regulation of metabolic process	3.060×10^{-7}	4.890×10^{-4}	173	858
GO:0065007	biological regulation	4.565×10^{-7}	7.294×10^{-4}	224	1343
GO:0023033	signaling pathway	5.167×10^{-7}	8.257×10^{-4}	73	315
GO:0043543	protein amino acid acylation	2.327×10^{-6}	0.00372	22	43
GO:0009987	cellular process	1.273×10^{-5}	0.02034	291	1997
GO:0051674	localization of cell	1.375×10^{-5}	0.02198	31	124
GO:0051276	chromosome organization	1.446×10^{-5}	0.02310	69	226
GO:0006383	transcription from RNA polymerase III promoter	1.914×10^{-5}	0.03058	10	11

Table 6 - Gene cluster 6 - GO enrichment analysis using Ontologizer [1] with settings "Parent-Child-Intersection/Bonferroni". For this analysis, a total of 2179 genes were in the population set, of which a total of 248 genes were in the study set.

ID	Name	p-Value	p-Value (Adj)	Study Count	Population Count
GO:0006414	translational elongation	4.689×10^{-29}	6.489×10^{-26}	83	83
GO:0006412	translation	1.062×10^{-22}	1.470×10^{-19}	89	133
GO:0019538	protein metabolic process	1.482×10^{-11}	2.051×10^{-8}	131	630
GO:0009058	biosynthetic process	8.929×10^{-10}	1.236×10^{-6}	155	915
GO:0042254	ribosome biogenesis	2.755×10^{-8}	3.813×10^{-5}	23	46
GO:0034660	ncRNA metabolic process	2.214×10^{-5}	0.03064	17	55
GO:0043170	macromolecule metabolic process	2.996×10^{-5}	0.04146	193	1392

Table 7 - Gene cluster 7 - GO enrichment analysis using Ontologizer [1] with settings "Parent-Child-Intersection/Bonferroni". For this analysis, a total of 2179 genes were in the population set, of which a total of 406 genes were in the study set.

ID	Name	p-Value	p-Value (Adj)	Study Count	Population Count
GO:0016071	mRNA metabolic process	1.688×10^{-78}	2.515×10^{-75}	179	204
GO:0006396	RNA processing	5.765×10^{-78}	8.589×10^{-75}	204	266
GO:0016070	RNA metabolic process	2.833×10^{-27}	4.221×10^{-24}	247	689
GO:0022613	ribonucleoprotein complex biogenesis	1.356×10^{-25}	2.020×10^{-22}	62	94
GO:0015931	nucleobase, nucleoside, nucleotide and nucleic acid transport	4.188×10^{-14}	6.240×10^{-11}	24	32
GO:0006403	RNA localization	2.943×10^{-12}	4.386×10^{-9}	24	32
GO:0008380	RNA splicing	1.113×10^{-10}	1.659×10^{-7}	166	189
GO:0010467	gene expression	1.238×10^{-10}	1.845×10^{-7}	265	958
GO:0006807	nitrogen compound metabolic process	5.475×10^{-10}	8.158×10^{-7}	269	1054
GO:0008152	metabolic process	2.237×10^{-9}	3.334×10^{-6}	335	1581
GO:0006401	RNA catabolic process	6.768×10^{-9}	1.008×10^{-5}	23	28
GO:0010608	posttranscriptional regulation of gene expression	1.193×10^{-8}	1.778×10^{-5}	28	70
GO:0043933	macromolecular complex subunit organization	1.271×10^{-8}	1.893×10^{-5}	71	287
GO:0006457	protein folding	3.811×10^{-8}	5.678×10^{-5}	17	31
GO:0043170	macromolecule metabolic process	1.830×10^{-7}	2.727×10^{-4}	320	1392
GO:0034621	cellular macromolecular complex subunit organization	5.032×10^{-7}	7.498×10^{-4}	54	147
GO:0042982	amyloid precursor protein metabolic process	1.858×10^{-5}	0.02768	6	7

Table 8 - Gene cluster 8 - GO enrichment analysis using Ontologizer [1] with settings "Parent-Child-Intersection/Bonferroni". For this analysis, a total of 2179 genes were in the population set, of which a total of 114 genes were in the study set.

ID	Name	p-Value	p-Value (Adj)	Study Count	Population Count
GO:0009058	biosynthetic process	4.476×10^{-18}	3.236×10^{-15}	95	915
GO:0006413	translational initiation	8.325×10^{-10}	6.019×10^{-7}	12	24
GO:0065007	biological regulation	1.141×10^{-7}	8.247×10^{-5}	96	1343
GO:0006996	organelle organization	4.596×10^{-7}	3.323×10^{-4}	44	405
GO:0010467	gene expression	9.042×10^{-7}	6.537×10^{-4}	89	958
GO:0043170	macromolecule metabolic process	1.894×10^{-6}	0.00137	100	1392
GO:0051276	chromosome organization	5.667×10^{-6}	0.00410	38	226
GO:0006476	protein amino acid deacetylation	2.349×10^{-5}	0.01699	5	10

Table 9 - Gene cluster 9 - GO enrichment analysis using Ontologizer [1] with settings "Parent-Child-Intersection/Bonferroni". For this analysis, a total of 2179 genes were in the population set, of which a total of 517 genes were in the study set.

ID	Name	p-Value	p-Value (Adj)	Study Count	Population Count
GO:0032501	multicellular organismal process	6.254×10^{-51}	1.453×10^{-47}	359	900
GO:0044281	small molecule metabolic process	3.815×10^{-33}	8.866×10^{-30}	113	223
GO:0065008	regulation of biological quality	8.025×10^{-26}	1.865×10^{-22}	176	408
GO:0050896	response to stimulus	6.438×10^{-23}	1.496×10^{-19}	278	773
GO:0006629	lipid metabolic process	1.093×10^{-19}	2.539×10^{-16}	63	121
GO:0003008	system process	1.078×10^{-18}	2.505×10^{-15}	195	332
GO:0005975	carbohydrate metabolic process	3.740×10^{-17}	8.691×10^{-14}	42	67
GO:0032502	developmental process	3.346×10^{-15}	7.776×10^{-12}	253	750
GO:0051179	localization	2.140×10^{-13}	4.973×10^{-10}	194	545
GO:0046483	heterocycle metabolic process	2.493×10^{-13}	5.794×10^{-10}	38	69
GO:0009308	amine metabolic process	3.906×10^{-13}	9.077×10^{-10}	30	53
GO:0044271	cellular nitrogen compound biosynthetic process	4.074×10^{-13}	9.468×10^{-10}	36	71
GO:0050953	sensory perception of light stimulus	1.810×10^{-10}	4.207×10^{-7}	68	81
GO:0051241	negative regulation of multicellular organismal process	2.261×10^{-9}	5.254×10^{-6}	34	51
GO:0043062	extracellular structure organization	3.500×10^{-9}	8.134×10^{-6}	31	56
GO:0010876	lipid localization	1.133×10^{-8}	2.633×10^{-5}	24	38
GO:0042157	lipoprotein metabolic process	1.347×10^{-8}	3.131×10^{-5}	13	18
GO:0007010	cytoskeleton organization	3.592×10^{-8}	8.347×10^{-5}	36	112
GO:0060415	muscle tissue morphogenesis	6.745×10^{-8}	1.567×10^{-4}	14	14
GO:0003007	heart morphogenesis	1.791×10^{-7}	4.161×10^{-4}	22	28
GO:0034367	macromolecular complex remodeling	2.066×10^{-7}	4.802×10^{-4}	10	11
GO:0009100	glycoprotein metabolic process	3.411×10^{-7}	7.927×10^{-4}	15	28
GO:0048878	chemical homeostasis	3.451×10^{-7}	8.021×10^{-4}	79	144
GO:0007154	cell communication	6.371×10^{-7}	0.00148	105	316
GO:0006725	cellular aromatic compound metabolic process	7.312×10^{-7}	0.00170	15	24
GO:0006518	peptide metabolic process	9.535×10^{-7}	0.00222	10	12
GO:0006091	generation of precursor metabolites and energy	9.561×10^{-7}	0.00222	27	63
GO:0007186	G-protein coupled receptor protein signaling pathway	2.018×10^{-6}	0.00469	37	81
GO:0003013	circulatory system process	2.879×10^{-6}	0.00669	63	78

ID	Name	p-Value	p-Value (Adj)	Study Count	Population Count
GO:0051336	regulation of hydrolase activity	3.925×10^{-6}	0.00912	38	90
GO:0006811	ion transport	3.982×10^{-6}	0.00926	72	130
GO:0009056	catabolic process	5.344×10^{-6}	0.01242	73	251
GO:0035150	regulation of tube size	7.447×10^{-6}	0.01731	24	32
GO:0007267	cell-cell signaling	7.976×10^{-6}	0.01854	67	146
GO:0055085	transmembrane transport	1.026×10^{-5}	0.02385	52	89
GO:0003205	cardiac chamber development	1.351×10^{-5}	0.03140	11	11
GO:0051604	protein maturation	1.936×10^{-5}	0.04499	15	39
GO:0050817	coagulation	1.987×10^{-5}	0.04617	29	40

Table 10 - Gene cluster 10 - GO enrichment analysis using Ontologizer [1] with settings "Parent-Child-Intersection/Bonferroni". For this analysis, a total of 2179 genes were in the population set, of which a total of 351 genes were in the study set.

ID	Name	p-Value	p-Value (Adj)	Study Count	Population Count
GO:0032501	multicellular organismal process	6.079×10^{-20}	1.273×10^{-16}	224	900
GO:0032502	developmental process	6.187×10^{-15}	1.295×10^{-11}	187	750
GO:0055114	oxidation reduction	1.783×10^{-9}	3.733×10^{-6}	29	63
GO:0007154	cell communication	6.421×10^{-9}	1.344×10^{-5}	87	316
GO:0006793	phosphorus metabolic process	8.839×10^{-9}	1.851×10^{-5}	70	270
GO:0006091	generation of precursor metabolites and energy	1.274×10^{-8}	2.667×10^{-5}	27	63
GO:0050954	sensory perception of mechanical stimulus	2.489×10^{-8}	5.213×10^{-5}	33	52
GO:0051604	protein maturation	3.564×10^{-8}	7.463×10^{-5}	16	39
GO:0044271	cellular nitrogen compound biosynthetic process	1.154×10^{-7}	2.417×10^{-4}	27	71
GO:0046483	heterocycle metabolic process	1.317×10^{-7}	2.758×10^{-4}	27	69
GO:0044281	small molecule metabolic process	1.505×10^{-7}	3.151×10^{-4}	61	223
GO:0002376	immune system process	5.929×10^{-7}	0.00124	73	266
GO:0023052	signaling	3.333×10^{-6}	0.00698	144	657
GO:0016265	death	3.500×10^{-6}	0.00733	86	343
GO:0007610	behavior	3.872×10^{-6}	0.00811	41	112
GO:0051179	localization	5.945×10^{-6}	0.01245	123	545
GO:0030030	cell projection organization	7.732×10^{-6}	0.01619	30	99

References

1. Bauer S, Grossmann S, Vingron M, Robinson PN: **Ontologizer 2.0–a multifunctional tool for GO term enrichment analysis and data exploration.** *Bioinformatics* 2008, **24**(14):1650–1651.
