# Supplementary figures and images for: Structuring heterogeneous biological information using fuzzy clustering of k-partite graphs
Source: BMC Bioinformatics. 2010 Oct 20;11:522. doi: 10.1186/1471-2105-11-522 (PMC3247861; doi:10.1186/1471-2105-11-522)

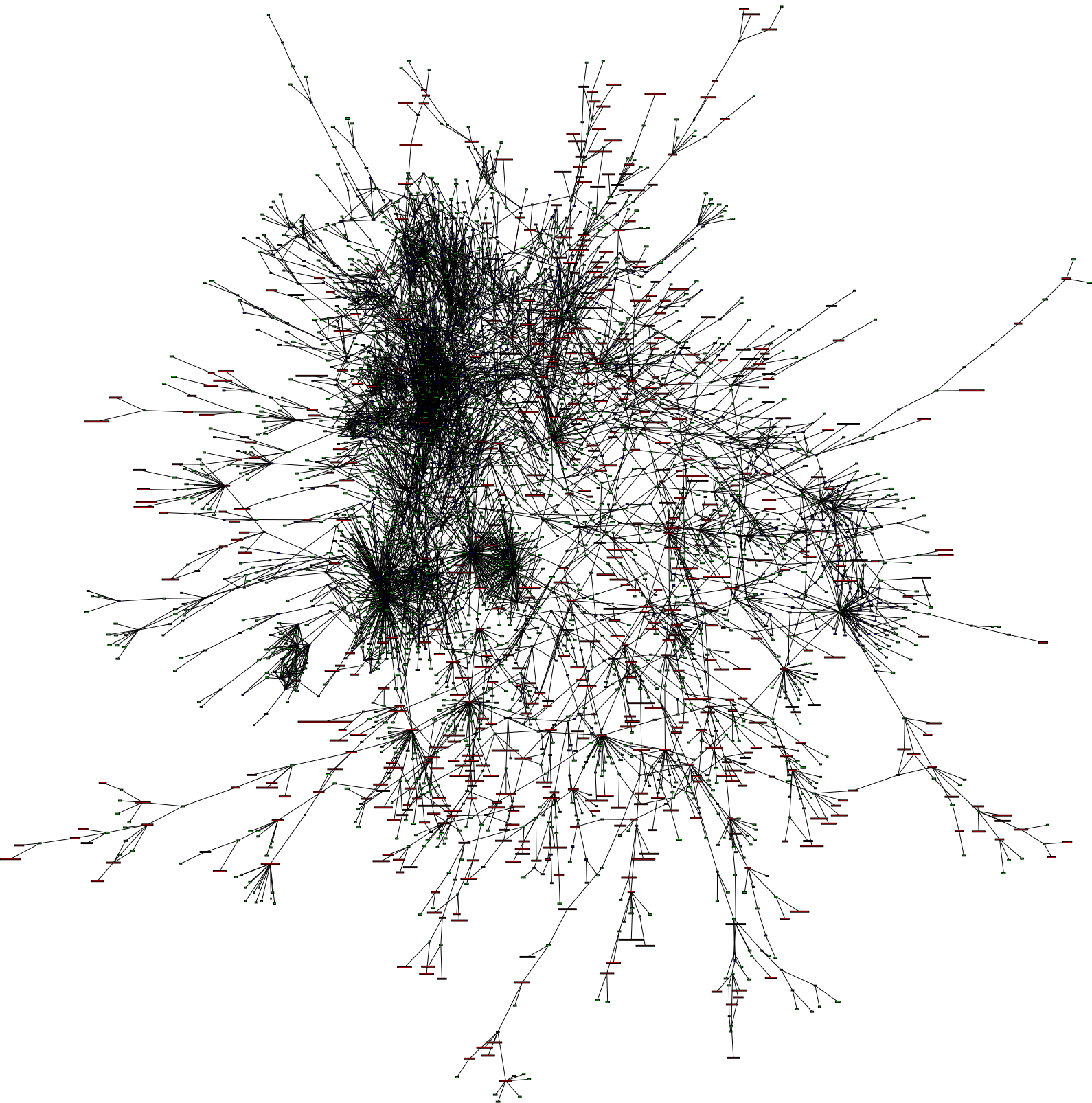

Supplement: Additional file 7 — Integrated tripartite network. Illustration of the largest connected component of the layered, tripartite graph gene-disease-protein complex network. It consists of 2293 gene (green), 590 disease (red) and 854 complex (blue) nodes connected by 6219 edges. [file 1471-2105-11-522-S7.PDF]
