# Supplementary material for: Structuring heterogeneous biological information using fuzzy clustering of k-partite graphs
Source: BMC Bioinformatics. 2010 Oct 20;11:522. doi: 10.1186/1471-2105-11-522 (PMC3247861; doi:10.1186/1471-2105-11-522)
Supplement: Additional 8 — FunCat and disorder class annotation tables. Table 1 shows the FunCat classes used for evaluating the gene and protein complex clusters. A subset of 13 FunCat main categories was taken from CORUM. Table 2 represents the 20 primary disorder classes retrieved from Goh et al. (2007). Additional classes are multiple, grey and unclassfied. [file 1471-2105-11-522-S8.PDF]

Tables

Table 1 shows the FunCat classes used for evaluating the gene and protein complex clusters. Table 2 represents the 20 primary disorder classes retrieved from Goh et al. (2007).

Table 1 - FunCat categories

A subset of 13 FunCat main categories was taken from CORUM to evaluate if identified clusters are biological feasible and if interconnected global communities are functionally correlated [1, 2].

FunCat ID	Description
02	Energy
10	Cell Cycle and DNA Processing
11	Transcription
12	Protein Synthesis
18	Regulation of Metabolism and Protein Function
20	Cellular Transport, Transport Facilities and Transport Routes
30	Cellular Communication/Signal Transduction Mechanism
32	Cell Rescue, Defense and Virulence
41	Development (systemic)
42	Biogenesis of Cellular Components
43	Cell Type Differentiation
45	Tissue Differentiation
47	Organ Differentiation

Table 2 - Disorder classes

20 primary disorder classes were retrieved Goh *et al.* [3]. Additional classes are *multiple, grey and unclassified.* For consistency reasons we added annotations from the *grey* category to the *multiple* class for the gene partition.

ID	Disorder
01	Bone
02	Cancer
03	Cardiovascular
04	Connective tissue
05	Dermatological
06	Development
07	Ear,Nose,Throat
08	Endocrine
09	Gastrointestinal
10	Hematological
11	Immunological
12	Metabolic
13	Multiple
14	Muscular
15	Neurological
16	Nutritional
17	Ophthamological
18	Psychiatric
19	Renal
20	Respiratory
21	Skeletal
22	Unclassified
23	Grey

References

1. Ruepp A, Zollner A, Maier D, Albermann K, Hani J, Mokrejs M, Tetko I, Güldener U, Mannhaupt G, Münsterkötter M, Mewes H: **The FunCat, a functional annotation scheme for systematic classification of proteins from whole genomes.** *Nucleic Acids Res* 2004, **32**(18):5539–5545.

2. Ruepp A, Brauner B, Dunger-Kaltenbach I, Frishman G, Montrone C, Stransky M, Waegele B, Schmidt T, Doudieu ON, Stümpflen V, Mewes H: **CORUM: the comprehensive resource of mammalian protein complexes.** *Nucleic Acids Res* 2008, **36**(Database issue):D646–D650.

3. Goh KI, Cusick ME, Valle D, Childs B, Vidal M, Barabási AL: **The human disease network.** *Proc Natl Acad Sci U S A* 2007, **104**(21):8685–8690.
